# Fast Marginal Likelihood Estimation of Penalties for Group-Adaptive Elastic Net

**DOI:** 10.1080/10618600.2022.2128809

**Published:** 2022-11-09

**Authors:** Mirrelijn M. van Nee, Tim van de Brug, Mark A. van de Wiel

**Affiliations:** Department of Epidemiology and Data Science, Amsterdam University Medical Centers, Amsterdam, The Netherlands

**Keywords:** Clinical prediction, Empirical Bayes, Omics, Penalized generalized linear models, Prior information

## Abstract

Elastic net penalization is widely used in high-dimensional prediction and variable selection settings. Auxiliary information on the variables, for example, groups of variables, is often available. Group-adaptive elastic net penalization exploits this information to potentially improve performance by estimating group penalties, thereby penalizing important groups of variables less than other groups. Estimating these group penalties is, however, hard due to the high dimension of the data. Existing methods are computationally expensive or not generic in the type of response. Here we present a fast method for estimation of group-adaptive elastic net penalties for generalized linear models. We first derive a low-dimensional representation of the Taylor approximation of the marginal likelihood for group-adaptive ridge penalties, to efficiently estimate these penalties. Then we show by using asymptotic normality of the linear predictors that this marginal likelihood approximates that of elastic net models. The ridge group penalties are then transformed to elastic net group penalties by matching the ridge prior variance to the elastic net prior variance as function of the group penalties. The method allows for overlapping groups and unpenalized variables, and is easily extended to other penalties. For a model-based simulation study and two cancer genomics applications we demonstrate a substantially decreased computation time and improved or matching performance compared to other methods. Supplementary materials for this article are available online.

## 1 Introduction

A popular approach to prediction and variable selection is elastic net penalization (Zou and Hastie 2005), as it simultaneously estimates and directly selects variables. Prediction and variable selection is hard for high-dimensional data, in which the number of variables far exceeds the number of samples. In addition to the main data, providing information on the samples, so-called co-data (van de Wiel et al. 2016) may be available, providing complementary information on the variables, for example, potentially overlapping groups of variables. These co-data may be exploited to improve the prediction and variable selection.

Early methods like group lasso (Meier, van de Geer, and Bühlmann 2008) or variations thereof (e.g., see Huang, Breheny, and Ma 2012 for a review) account for group structure by selecting entire groups with the level of regularization governed by one global penalty parameter. While this may be useful when there are many small groups, its limited flexibility may render inferior predictive performance for settings with a limited number of groups differing largely in prediction strength (Münch et al. 2019). More recent work includes co-data by allowing for differential group penalty parameters, thereby flexibly penalizing informative groups of variables less than non-informative ones. The full Bayesian analogue of this would be to impose group-specific shrinkage priors with a hyperprior on the group prior parameters. The latter, however, shows already for the basic elastic net a high sensitivity of the results to the specification of the hyperprior parameters (Li and Lin 2010). Instead, empirical Bayes approaches estimate group penalties from the data and show competitive results to full Bayesian approaches for high-dimensional data (van de Wiel, Te Beest, and Münch 2019). The empirical Bayes EM algorithm proposed in Li and Lin (2010) does not allow for multiple groups of variables and -like the full Bayes alternative- does not scale in the number of variables, as the Gibbs sampler is computationally expensive for high-dimensional data. The method ipflasso (Boulesteix et al. 2017) uses cross-validation to select the best group penalties from a proposed grid of possible values. In general, however, it is unclear what values should be proposed. Moreover, the method does not scale in the number of groups as the number of possible grid configurations increases exponentially. The method gren (Münch et al. 2019), for group-adaptive elastic net, uses a variational Bayes algorithm to compute empirical Bayes estimates for the group penalties in logistic regression. The method is group-adaptive and scalable in the number of groups, but not in the number of variables. Moreover, it is only implemented for binary response. The method fwelnet (Tay et al. 2020) extends use of grouped co-data to continuous co-data (termed features of features). The method may be used for grouped co-data as well, for which it is shown to correspond to an elastic net penalty on the group level, with the amount of penalization governed by one global penalty parameter. Though fast, this method may, therefore, not be flexible enough. Finally, other group-adaptive methods have been developed specifically for priors other than the elastic net, for example, for spike-and-slab (Tang et al. 2018; Velten and Huber 2019) and ridge (van Nee, Wessels, and van de Wiel 2021; van de Wiel, van Nee, and Rauschenberger 2021).

Here we propose a fast and efficient method for marginal likelihood estimation of group elastic net penalties. The method is group-adaptive and may be used for elastic net penalized generalized linear models in high-dimensional data. Notably, its use is limited to high-dimensional data, as its applicability to low-dimensional data (with a small number of variables) has not been proven. We include details for linear and logistic regression. Groups may be overlapping and the method allows for unpenalized variables, such as an intercept or low-dimensional baseline variables like age and sex. First, we derive a low-dimensional representation of the marginal likelihood for ridge models, a special case of elastic net that does not select variables. Then we show by using the asymptotic multivariate normality of the linear predictor that this marginal likelihood also approximates the marginal likelihood of elastic net models well. We include a practical check for this multivariate normality assumption. Finally, we show how the ridge group penalties are transformed to find optimal marginal likelihood group penalties for elastic net models that select variables, to accommodate medium sparse to dense models. Besides its main contribution of efficiently estimating group-adaptive elastic net penalties, our method has the additional advantage that it is easily extended for estimation of group-adaptive penalties other than the elastic net ones.

The outline of the article is as follows. Section 2 includes details of the method. Section 3 compares performance and computation times of several methods in a linear regression model-based simulation study. Then, it illustrates the benefit of learning from co-data for variable selection. Finally, the method is illustrated in the logistic regression setting on two cancer genomics data examples. Section 4 discusses the method and results.

## 2 Method

Let the response be given by $Y\in R^{n}$ and the observed high-dimensional data, *p* > *n*, by $X\in R^{n\times p}$ . Suppose that the co-data provide *G* < *n* groups of variables. First assume that groups are not overlapping. Section 2.4 discusses how the method may be used for partly overlapping groups too. Let the observed data for group *g* be given by *X_g_*. We model the response with a generalized linear model (glm) with corresponding canonical link function g(·) , mean $\mu_{i}:=E_{Y_{i}|X_{i},\beta ,\phi }(Y_{i})=g^{-1}(X_{i}\beta )$ and variance $\text{var}(Y_{i})=\phi V(\mu_{i})$ for some specific variance function V(·) and scale parameter ϕ (McCullagh and Nelder 1989). Besides, we impose a conditional group-regularized elastic net prior on the regression coefficients $\beta \in R^{p}$ , with group penalties $\lambda \in R^{G}$ :

(1) $$\begin{array}{c} \pi \left(Y_{i}|X_{i},\beta ,\phi \right)\overset{\text{ind}.}{=}\;\exp\;(\frac{1}{\phi }(Y_{i}X_{i}\beta \\ \;-b(X_{i}\beta ))+c(Y_{i},\phi )),\;i=1,\ldots ,n,\\ \pi (\beta_{k}|\alpha ,\lambda_{g_{k}},\phi )\overset{\text{ind}.}{\propto }\;\exp\;(-\frac{\lambda_{g_{k}}}{2\phi }(\alpha |\beta_{k}|\\ \;+(1-\alpha )\beta_{k}^{2})),\;k=1,\ldots ,p, \end{array}$$

with b(·) and c(·) some specific functions corresponding to the type of generalized linear model used, and $\lambda_{g_{k}}$ the group-specific penalty of group *g* to which variable *k* belongs. The group-specific penalties are scaled by ϕ as in Li and Lin (2010) such that the penalized maximum likelihood estimate for β does not depend on ϕ (Equation (3)). Note that the group-adaptive prior only depends on ϕ and λ via the scaled inverse penalties defined as $\tau^{2}:=\phi /\lambda$ . Below, we consider *X* and α fixed. Selection of α is discussed in Section 2.3.1.

We use an empirical Bayes approach and estimate the group penalties and scale parameter for a given α to arrive at the group-adaptive regularized elastic net estimate for the regression coefficients:

(2) $$\begin{array}{c} \left(\hat{\lambda },\hat{\phi })=\underset{\lambda ,\phi }{\text{argmax}}\pi (Y|\alpha ,\lambda ,\phi \right)\\ =\underset{\lambda ,\phi }{\text{argmax}}\int_{\beta }\pi (Y|\beta ,\phi )\pi (\beta |\alpha ,\lambda ,\phi )d\beta , \end{array}$$

(3) $$\begin{array}{c} \hat{\beta }=\underset{\beta }{\text{argmax}}\left\{\;\log\;(\pi (Y|\beta ,\hat{\phi }))+\;\log\;(\pi (\beta |\alpha ,\hat{\lambda },\hat{\phi }))\right\}\\ =\underset{\beta }{\text{argmax}}\{\sum_{i=1}^{n}\left[Y_{i}X_{i}\beta -b(X_{i}\beta )\right]\\ \;+\sum_{k=1}^{p}\left[-\frac{{\hat{\lambda }}_{g_{k}}}{2}(\alpha |\beta_{k}|+(1-\alpha )\beta_{k}^{2})\right]\}. \end{array}$$

The data *X* possibly contain some unpenalized variables next to the penalized variables, for example, for an intercept, which we will refer to by $X_{\text{unpen}}\in R^{n\times p_{1}}$ and $X_{\text{pen}}\in R^{n\times p_{2}}$ , respectively, $p=p_{1}+p_{2}$ . In that case, only the penalized variables are integrated out in Equation (2). We refer to the unpenalized and penalized regression coefficients by $\beta_{\text{unpen}}$ and $\beta_{\text{pen}}$ .

### 2.1 Fast Marginal Likelihood Estimation for Group-Adaptive Ridge

First, consider group-regularized ridge models (*α* = 0), as we need those results for the general elastic net setting. Wood (2011) derives a Laplace approximation for the marginal likelihood for semiparametric generalized linear models when the prior is of the form:

(4) $$\beta |\lambda ,\phi ∼N(0,\phi S^{-}),\;S=\sum_{g}\lambda_{g}S_{g},$$

with *S*^–^ a generalized inverse and *S* the ridge penalty matrix equal to the weighted sum over *G* different known, possibly full penalty matrices *S_g_*. The R-package mgcv implementing this approximation is developed for low-dimensional data and does not allow for high-dimensional data. In theory, the results include the special case of group-adaptive ridge models in high-dimensional data as well, that is, substitute *S_g_* by ${\tilde{I}}_{g}\in R^{p\times p}$ , with ${\tilde{I}}_{g}$ the diagonal matrix with the *k*th diagonal element equal to 1 if variable *k* belongs to group *g* and 0 otherwise, and substitute *S* by $\Lambda :=\sum_{g}\lambda_{g}{\tilde{I}}_{g}$ as the corresponding group-adaptive ridge, diagonal penalty matrix. In practice, however, the Laplace approximation may be inaccurate for high-dimensional integrals (van de Wiel, Te Beest, and Münch 2019) and is computationally expensive due to the large dimension of *p*.

For ridge models, we may rewrite the high, *p*-dimensional integral as a lower, *n*-dimensional integral by observing that the likelihood only depends on β via the linear predictors η=Xβ (Veerman, Leday, and van de Wiel 2019). The resulting prior distribution for $\eta_{\text{pen}}=X_{\text{pen}}\beta_{\text{pen}}\in R^{n}$ is again a multivariate normal distribution:

(5) $$\eta_{\text{pen}}|\lambda ,\phi ∼N\left(0,X_{\text{pen}}\phi \Lambda_{\text{pen}}^{-1}X_{\text{pen}}^{T}\right)=N\left(0,\sum_{g}\tau_{g}^{2}X_{g}X_{g}^{T}\right),$$

with $\Lambda_{\text{pen}}\in R^{p_{2}\times p_{2}}$ denoting the diagonal ridge penalty matrix for the penalized variables.

Here, we derive a low-dimensional representation of the high-dimensional Laplace approximated marginal likelihood and its first derivative as derived by Wood (2011). We adapt it so that it is computed efficiently for multiple groups and allows for inclusion of unpenalized variables. This adaptation exploits results from van de Wiel, van Nee, and Rauschenberger (2021), who show that the maximum penalized likelihood estimate for the linear predictors and for a given $\lambda ,\;\hat{\eta }(\lambda )$ , may be obtained efficiently by rewriting the steps of the iterative weighted least squares algorithm (IWLS) in *n*-dimensional terms. We then use a general purpose optimizer to optimize the marginal likelihood for group-regularized ridge models. Below we state the results, details are given in Section A, supplementary materials. Note that subsequently, we use the shorthand notation $\hat{\eta }$ and drop the dependence on λ .

The Laplace approximation of the negative log marginal likelihood for group-regularized ridge models is

(6) $$-l(\lambda ,\phi )\approx -l(\hat{\eta },\phi )+\frac{1}{2\phi }{\left(y-\mu \right)}^{T}\hat{\eta }-\frac{1}{2}\log\;(|I_{n\times n}-WH_{\text{pen}}|),$$

with l(c) denoting the log likelihood given parameters ***c***, $I_{n\times n}\in R^{n\times n}$ the identity matrix, $W\in R^{n\times n}$ the weight matrix in IWLS, and *H_pen_* the hat matrix for the penalized variables:

(7) $$H_{\text{pen}}=X_{\text{pen}}{\left(X_{\text{pen}}^{T}WX_{\text{pen}}+\Lambda_{\text{pen}}\right)}^{-1}X_{\text{pen}}^{T}.$$

We state the derivative in terms of $\rho_{g}=\;\log\;(\lambda_{g})$ . First, denote the full hat matrix by *H*, that is, as in Equation (7) but with *X_pen_* substituted by *X*, and denote the contribution of the *g*th group to the hat matrix by $H_{g}:=X{\left(X^{T}WX+\Lambda \right)}^{-1}I_{g}X^{T}$ , with the efficient lower-dimensional representation given in Section A, Supplementary materials. The partial derivatives of the Laplace approximation of the log marginal likelihood to the group parameters are:

(8) $$\begin{array}{c} \frac{\partial \left(-l(\rho ,\phi )\right)}{\partial \rho_{g}}\\ =\frac{1}{\phi }{\left(y-\mu \right)}^{T}V^{-1}G\prime^{-1}\left[I_{n\times n}-HW\right]H_{g}^{T}\left(y-\mu +W\hat{\eta }\right)\\ \;-\frac{1}{2\phi }{\left(-G\prime^{-1}\hat{\eta }+y-\mu \right)}^{T}\left[I_{n\times n}-HW\right]H_{g}^{T}\left(y-\mu +W\hat{\eta }\right)\\ \;+\frac{1}{2}\text{tr}\left(H_{\text{pen}}\frac{\partial W}{\partial \rho_{g}}\right)\\ \;-\frac{1}{2}\text{tr}\left(\;\exp\;(-\rho_{g})X_{g}X_{g}^{T}{\left(W^{-1}+\sum_{g=1}^{G}\;\exp\;(-\rho_{g})X_{g}X_{g}^{T}\right)}^{-1}\right). \end{array}$$

where $V\in R^{n\times n}$ is the diagonal matrix with diagonal elements $V(\mu_{i}),\;G\prime$ is the diagonal matrix with diagonal elements $g\prime (\mu_{i})$ , and the partial derivative $\frac{\partial W}{\partial \rho_{g}}$ readily obtained from Wood (2011). The chain rule implies that the partial derivatives for *λ_g_* are obtained by multiplying the derivative to *ρ_g_* by $\frac{1}{\lambda_{g}}$ .

We optimize ϕ jointly with the group parameters. As $\hat{\eta }$ does not depend on ϕ , the partial derivative is given by:

(9) $$\frac{\partial \left(-l(\rho ,\phi )\right)}{\partial \phi }=\frac{\partial \left(-l(\hat{\eta },\phi )\right)}{\partial \phi }-\frac{1}{2\phi^{2}}{\left(y-\mu \right)}^{T}\hat{\eta }.$$

The partial derivative of the log likelihood depends on the type of glm considered and is known for canonical models. We include details for linear regression in Section A, supplementary materials. For logistic regression, ϕ=1 is constant and the partial derivative is 0.

### 2.2 Asymptotic Normality of the Linear Predictors

Next, we show that the linear predictors are asymptotically multivariate normal, corresponding to a group-adaptive ridge prior. The marginal likelihood for elastic net models is then shown to be approximately equal to a reparameterization of the ridge marginal likelihood for which we just showed how to compute (and optimize) it very efficiently.

Without loss of generality, consider the penalized variables only and leave out subscripts $\cdot_{pen}$ for notational convenience. Denote the ridge penalty parameters, penalty matrix and scaled ridge prior variances by $\lambda_{R}$ , Λ*_R_* and $\tau_{R}^{2}=\phi /\lambda_{R}$ respectively. Define the variance function of the elastic net prior distribution for a given α∈[0,1] as h(·) :

(10) $$h(\tau_{g_{k}}^{2})=h(\phi \lambda_{g_{k}}^{-1}):={\text{var}}_{\beta |\alpha ,\lambda ,\phi }(\beta_{k}).$$

Note that for each $\tau_{g_{k}}^{2},\;g_{k}\in \{1,\ldots ,G\}$ , there exist $\tau_{R,g}^{2}$ such that $h(\tau_{g_{k}}^{2})=\tau_{R,g}^{2}$ .

The prior distribution for η=Xβ is not analytical for α>0 . An important observation is that we can exploit the high-dimensionality of the data to approximate the prior for η with a multivariate normal distribution. The following theorem follows from the multivariate central limit theorem for linear random vector forms in Eicker (1966):

Theorem 1. Suppose $\beta_{j}\overset{\text{ind}.}{∼}\pi_{g_{j}}(\beta_{j})$ for j=1,…,p and group-specific prior $\pi_{g_{j}}(\cdot )$ , with $E(\beta_{j})=0$ and $\text{var}(\beta_{j})=\tau_{R,g_{j}}^{2}\in (0,\infty )$ . For g=1,…,G , let *G_g_* be the group size and let $X_{g}\in R^{n\times G_{g}}$ be the observed data corresponding to group *g*. Let $x_{*j}$ denote the *j*th column of $X\in R^{n\times p}$ . Suppose x*j=0∈Rn for all *j*, rank(*X*) = *n* for all *p*, and for p→∞ ,

(11) $$\max_{j=1,\ldots ,p}x_{*j}^{T}{\left(XX^{T}\right)}^{-1}x_{*j}\to 0.$$

Then, for fixed *G*, fixed *n*, and p→∞ ,

(12) $${\left(\sum_{g}\tau_{R,g}^{2}X_{g}X_{g}^{T}\right)}^{-1/2}X\beta \overset{d}{\to }N(0,I_{n\times n}),$$

where $I_{n\times n}$ is the (n×n) -dimensional identity matrix and ${\left(\sum_{g}\tau_{R,g}^{2}X_{g}X_{g}^{T}\right)}^{-1/2}$ is the inverse of the unique positive definite square root of $\sum_{g}\tau_{R,g}^{2}X_{g}X_{g}^{T}$ .

**Fig. 1:**
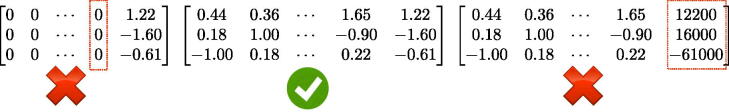
Illustration of the assumptions for the data matrix *X* in Theorem 1: zero columns are not allowed (left) nor variables that dominate the other variables (right). The visual check described in Section 2.6 may be used to check whether the multivariate normality assumption of the linear predictors is reasonable for the data and prior.

If *n* = 1 then condition (11) is equivalent to $\max_{j=1,\ldots ,p}x_{1j}^{2}/\sum_{j=1}^{p}x_{1j}^{2}\to 0$ , for p→∞ . Informally, condition (11) can be interpreted as each variable being asymptotically negligible in size compared to the full dataset, illustrated in Figure 1. In practice, this condition will be reasonable for most high-dimensional data, especially as these data are often standardized, but counter examples may exist such as data with extreme outliers.

Hence, the prior on η under an elastic net prior on β may be approximated by the following multivariate normal distribution for $\tau_{R,g}^{2}$ such that $\tau_{R,g}^{2}=h(\tau_{g}^{2})$ for each *g*:

(13) $$\begin{array}{c} \pi (\eta |\alpha ,\lambda ,\phi )\approx N\left(0,\sum_{g}h(\tau_{g}^{2})X_{g}X_{g}^{T}\right)\\ =N\left(0,\sum_{g}\tau_{R,g}^{2}X_{g}X_{g}^{T}\right)=\pi (\eta |\lambda_{R},\phi ). \end{array}$$

Then, for $\lambda_{R,g}$ corresponding to $\tau_{R,g}^{2}$ such that $\tau_{R,g}^{2}=h(\tau_{g}^{2})$ , the marginal likelihood may be approximated as follows:

(14) $$\begin{array}{c} \pi (Y|\alpha ,\lambda ,\phi )=\int_{\eta }\pi (Y|\eta ,\phi )\pi (\eta |\alpha ,\lambda ,\phi )d\eta \\ \approx \int_{\eta }\pi (Y|\eta ,\phi )\pi (\eta |\lambda_{R},\phi )d\eta =\pi (Y|\lambda_{R},\phi ), \end{array}$$

where the latter expression is efficiently computed using Equation (6). Hence, the marginal likelihood of group-regularized elastic net models is approximately the same as that of ridge models, but parameterized differently. The partial derivatives from the elastic net parameterization may then be obtained from the partial derivatives for the ridge parameterization as given in Equations (8) and (9) by using the chain rule and a change of variables.

### 2.3 Fast Marginal Likelihood Estimation for Group-Adaptive Elastic Net

**Fig. 2:**
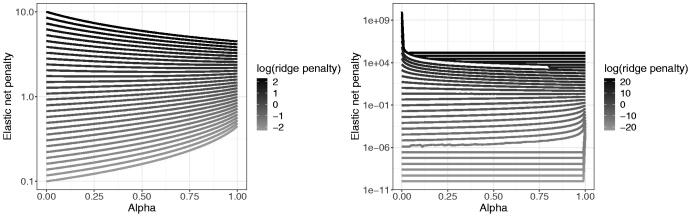
Transformed elastic net penalty for various α and ridge penalties.

As we desire variable selection, we would like to obtain group-adaptive elastic penalties for other cases (0<α≤1 ) than the ridge model (*α* = 0). Given the approximation for the marginal likelihood in Equation (14), one could again use a general purpose optimizer to maximize the marginal likelihood for the elastic net parameterization. We, however, propose to use an indirect approach and simply transform the marginal likelihood estimates for the ridge parameterization to the elastic net parameterization using the known variance function. As the marginal likelihood of the ridge and elastic net model are approximately equal, the maximal value, obtained in the transformed maximizer, is also approximately equal. So, the elastic net estimates are given by

(15) $${\hat{\tau }}^{2}=h^{-1}\left({\hat{\tau }}_{R}^{2}\right),\;{\hat{\lambda }}_{g}=\hat{\phi }/{\hat{\tau }}_{g}^{2},\;g=1,\ldots ,G,$$

where $h^{-1}(\cdot )$ is applied element-wise. The proposed approach has the advantage that, once the optimal ridge penalties are obtained, the optimal elastic net penalties are quickly obtained for a whole range of possible α values. Figure 2 illustrates this.

The variance function for the elastic net prior for a given α is

(16) $$\begin{array}{c} h(\tau_{g}^{2})={\text{var}}_{\beta |\alpha ,\lambda_{g},\phi }(\beta_{k})=\tau_{g}^{2}{\left(1-\alpha \right)}^{-1}\\ \;-\frac{\alpha \tau_{g}}{2{\left(1-\alpha \right)}^{3/2}}\frac{\varphi \left(\frac{\alpha }{2\tau_{g}{\left(1-\alpha \right)}^{\frac{1}{2}}}\right)}{1-\Phi \left(\frac{\alpha }{2\tau_{g}{\left(1-\alpha \right)}^{\frac{1}{2}}}\right)}+\frac{\alpha^{2}}{4{\left(1-\alpha \right)}^{2}}, \end{array}$$

with Φ(·) and φ(·) the standard normal cumulative density function and probability density function respectively.

The expression simplifies for lasso (*α* = 1) to $h(\tau_{g}^{2})=8\tau_{g}^{4}$ , and is easily solved analytically. For 0<α<1 , we use the root-finding algorithm of the R-function uniroot to find the roots of $h(\tau_{g}^{2})-{\hat{\tau }}_{R,g}^{2}=0$ . This suffices for values of $10^{-6}<\tau_{g}^{2}<10^{6}$ . The evaluation of the variance function is numerically unstable for more extreme values of $\tau_{g}^{2}$ . Therefore, we truncate the values to either the ridge or lasso estimate while maintaining the monotonicity for more extreme $\tau_{g}^{2}$ , as illustrated in Figure 2. We expect that this fix will suffice as the precise absolute value has less impact in the extremes.

#### Practical Selection of α

2.3.1

Joint optimization of α and one elastic net penalty *λ* is hard as the marginal likelihood shows a flat optimal region (van de Wiel, Te Beest, and Münch 2019). For our approach specifically, the approximated marginal likelihood is even identical for all α and transformed λ (Equation (14)), making a jointly optimal α unidentifiable. Stacked elastic net (Rauschenberger, Glaab, and van de Wiel 2020) may improve elastic net performance by combining multiple values of α instead of selecting one value, but does not produce sparse models. In practice, we advise to compare the cross-validated likelihood or another performance criterion for few, say four or five, fixed α -values ranging from 0 to 1. One may then select the model with the best performance or the sparsest model with similar performance. In our application in Section 3.2, for example, we compared the cross-validated AUC performance of models for α∈{0.01,0.3,0.8,1} .

### 2.4 Overlapping Groups

So far, we have assumed the groups to be nonoverlapping. A simple way to allow for partly overlapping groups is to make artificial, nonoverlapping groups, similarly as proposed as naive implementation of the latent overlapping group lasso (Jacob, Obozinski, and Vert 2009). Full details are included in Section A.5, supplementary materials. In short, columns of *X* of variables that belong to multiple groups are first duplicated and scaled such that the corresponding prior on the linear predictors is identical to the one corresponding to the original data, to account for multiplicity. This results in the extended data matrix $\tilde{X}\prime$ with artifical nonoverlapping groups, on which our method may be used to obtain the group-adaptive penalty estimates. The dimension of $\tilde{X}\prime$ increases rapidly for largely overlapping groups. In practice, however, it is not necessary to store this matrix as the computations for the marginal likelihood require the high-dimensional computation of *G n* × *n*-dimensional matrices $\tilde{X}\prime_{g}\tilde{X}\prime_{g}{}^{T}$ only once. Finally, given the penalty estimates, the original data matrix *X* is used to obtain estimates for the regression coefficients.

### 2.5 Recalibration by Cross-validation

In particular for logistic regression, we experienced that the components *λ_g_* of λ are estimated well in a relative sense, but less so in the absolute sense: penalties are overestimated, leading to overpenalized regression coefficients and sub-optimal prediction performances (exemplified in Section B.1.1 in the supplementary materials). While in practice the Laplace approximation is accurate enough to be applied to a wide range of distributions (Rue, Martino, and Chopin 2009), the problem of overpenalization is known to arise when the marginal likelihood is approximated for “difficult” binary responses (Kuss, Rasmussen, and Herbrich 2005; Ferkingstad and Rue 2015). The overpenalization does not show for Poisson distributed response or binomial response with more than one repeated observation (Veerman, Leday, and van de Wiel 2019). Initial errors in the ridge penalty maximizers may be amplified when transforming to elastic net penalties (see Figure 6).

Though more accurate, but more complex approximations may improve the estimates (Ferkingstad and Rue 2015; Kuss, Rasmussen, and Herbrich 2005), here we propose to counter the overpenalization with a simple approach of recalibrating the group penalties with one cross-validated global rescaling penalty, *λ*_0_: $\lambda \prime =\lambda_{0}\lambda$ . So, after estimating the group penalties λ by maximizing the approximate marginal likelihood, we estimate *λ*_0_ by cross-validation of the likelihood. As *λ*_0_ is a scalar, this can efficiently and easily be done, for example, by using glmnet with penalty factors λ . Whether or not the recalibration step is needed for generalized linear models other than for linear and logistic response may in practice be checked by comparing the prediction performance of the models with and without recalibration.

### 2.6 Normality Check

Our hyperparameter estimation relies on the assumption of multivariate normality of η=Xβ, where β is generated from the prior. A posteriori, this assumption is easily checked by repeatedly sampling β from its estimated (elastic net) prior, rendering $\beta^{\left(k\right)}$ , and computing $\eta^{\left(k\right)}=X\beta^{\left(k\right)}$ , for k=1,…,K . Then, compute the Mahalanobis distances

$$d^{\left(k\right)}={\left(\eta^{\left(k\right)}-\mu \right)}^{T}\Sigma^{-1}(\eta^{\left(k\right)}-\mu ),$$

where μ=0 , because the prior has mean zero, and covariance matrix $\Sigma =\phi \sum_{g}\lambda_{R,g}^{-1}X_{g}X_{g}^{T}$ as in Equation (5). Finally, we propose the standard visual check for multivariate normality by a QQ-plot: the empirical quantiles of $d={\left(d^{\left(k\right)}\right)}_{k=1}^{K}$ are plotted against the theoretical ones of the $\chi_{n}^{2}$ distribution. Figure A1, supplementary materials shows some examples for different priors. The normality check is accommodated by the software.

### 2.7 Extension to other Sparse Priors

The method may easily be extended to other group-adaptive sparse penalties corresponding to group-specific priors with finite variance. For any such prior, we only need to know its variance function and invert this (numerically) to find the marginal likelihood estimates for the group-specific prior parameters as in Equation (15). We provide examples in Section A.7, supplementary materials for two other well-known priors; a spike-and-slab prior and the generalized normal prior, corresponding to the bridge penalty.

## 3 Data Examples

The method is termed squeezy as it squeezes out some sparsity from dense group-adaptive ridge models by transforming ridge penalties to sparse penalties. We conduct a model-based linear regression simulation study and illustrate the method on two cancer genomics examples in the logistic regression setting. We include the following elastic net models, summarized in Table 1, to compare performance and computation time:

**Table 1:** Overview of the methods compared in Section 3.

Method	glmnet	fwelnet	ipflasso	gren	**multiridge** ${}^{*}$	ecpc	squeezy
Group-adaptive	x	x	x/v ${}^{†}$	v	v	v	v
Group parameter estimation	NA	Joint optimisation	CV	Variational Bayes EM	Fast CV	Marginal moment	Marginal likelihood
Elastic net	v	v	v	v	x (only ridge)	x (only ridge)	v
GLM	v	v	v	x (only binomial)	v	v	v

NOTE: *Multiridge (van de Wiel, van Nee, and Rauschenberger 2021) is not included in the data examples, but some of its computational shortcuts are used in squeezy as explained in Section 2.1. ^†^ Ipflasso is able to adapt group penalties, but for a finite set of proposed values only.

1. EN (glmnet, Friedman, Hastie, and Tibshirani 2010): a co-data agnostic elastic net penalty, with global penalty parameter obtained by cross-validation (CV);
2. fwEN and fwEN (continuous) (fwelnet, Tay et al. 2020): a globally adaptive elastic net penalty on group level for grouped co-data (fwEN) or elastic net penalty with weights a function of continuous co-data (fwEN (continuous)). Note that we include fwelnet (continuous) only in the first cancer genomics data example for which continuous co-data is available;
3. ipf and ipf2 (ipflasso, Boulesteix et al. 2017): a group-adaptive elastic net penalty, which uses cross-validation to select the group penalty factors from a grid of possible values. We take the grid where each penalty factor is in {1, 2} (ipf) or in {1, 2, 4} (ipf2). Note that we only include the computationally expensive ipf2 on the whole dataset in the model-based simulated data example for comparison of computing times;
4. gren (gren, Münch et al. 2019): a group-adaptive elastic net penalty, with the group penalty factors obtained by an approximate empirical-variational Bayes framework. Note that gren is not included in the first linear regression example, as it is not implemented for the linear model;
5. ecpcEN squeezy (ecpc, van Nee, Wessels, and van de Wiel 2021 and squeezy): a group-adaptive elastic net penalty. The method ecpc provides empirical Bayes moment estimates for group-adaptive ridge penalties. We use squeezy to transform these to elastic net penalties combined with a recalibrated global rescaling penalty;
6. squeezy (single) and squeezy (single + reCV): a co-data agnostic elastic net penalty, where the global penalty is obtained by transforming the marginal likelihood estimate for the ridge penalty to an elastic net penalty, without or with recalibration of a global rescaling penalty. While glmnet internally standardizes linear response, squeezy (single + reCV) does not;
7. squeezy (multi) and squeezy (multi + reCV): a group-adaptive elastic net penalty using the proposed method, without or with recalibration of a global rescaling penalty.

### 3.1 Model-based Simulation Study

We perform a model-based simulation study to compare prediction performance and computation time of several methods, and to compare group parameter estimates with known sample estimates. We show that our method improves upon co-data agnostic methods by learning from informative co-data, that is, estimating differential group priors, and scales better than other group-adaptive methods. Finally, we illustrate the benefit of learning from co-data for the purpose of variable selection in two other model-based simulated scenarios.

We simulate training and test sets of observed data *X* in 10 correlated blocks of size p/10 , regression coefficients from a Laplace distribution (*α* = 1) with mean zero and group-specific scale parameter $b_{g_{k}},\;g_{k}\in \{1,\ldots ,5\}$ , for five equally sized groups, and response from a normal distribution:

$$\begin{array}{c} x_{i,b}\overset{\text{ind}.}{∼}N\left(0,I_{\left(p/10)\times (p/10\right)}+\frac{\rho^{2}}{{\left(1-\rho \right)}^{2}}1_{\left(p/10)\times (p/10\right)}\right),\\ \;i=1,\ldots ,n,\;b=1,\ldots ,10,\\ \beta_{k}\overset{\text{ind}.}{∼}\text{Laplace}(0,b_{k_{g}}),\;k=1,\ldots ,p,\;Y∼N(X\beta ,\sigma^{2}I_{n\times n}), \end{array}$$

with ρ=0.2 and $\sigma^{2}=2$ . A similar simulation study for binary response is included in Section B.1.1 in the supplementary materials. We consider three settings of group parameters: (i) no groups: the group scale parameters ***b*** are the same and chosen such that the variance under each group prior is equal to $(0.1,0.1,0.1,0.1,0.1)\cdot \frac{1200}{p}$ ; (ii) weakly informative groups: the group scale parameters are different, but less so than in the third setting. The variances match $(0.141,0.161,0.186,0.336,0.536)\cdot \frac{1200}{p}$ ; (iii) informative groups: groups are different, with variances matching $(0.01,0.05,0.1,0.4,0.8)\cdot \frac{1200}{p}$ .

#### 3.1.1 Prediction Performance Comparison

**Fig. 3:**
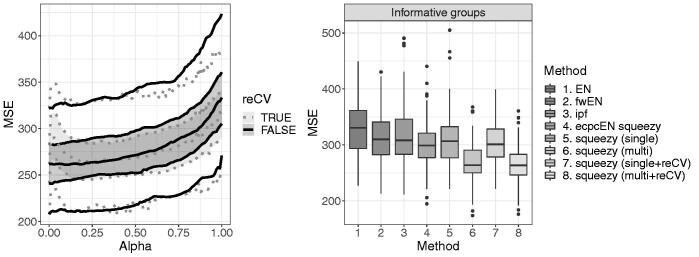
Model-based simulation study, informative groups setting. Left: MSE performance of squeezy (multi) and squeezy (multi + reCV) on the test sets of 100 pairs of training and test sets for 100 evenly spaced values of α∈[0,1] , shown in pointwise quantiles of (0.05,0.25,0.5,0.75,0.95) . Right: boxplots of the MSE performance on the test sets of 100 pairs of training and test sets, α=0.3 .

First, we fit the methods listed above for α=0.3 on 100 independent training and test sets, with *n* = 150, *p* = 1200 and the co-data providing group membership of the *G* = 5 groups, to compare performance. Our method squeezy easily obtains optimal elastic net group penalties for a range of α . We observe that there is usually little benefit from recalibration by cross-validation (reCV) in the linear case (Figure 3).

**Fig. 4:**
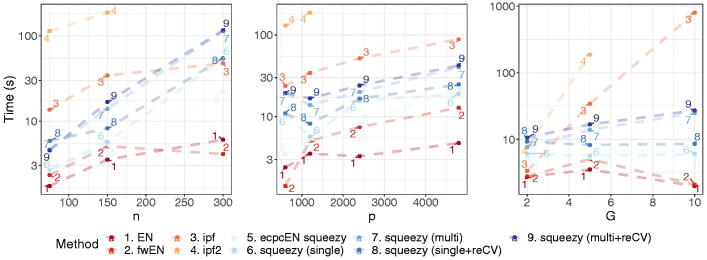
Model-based simulation study. Average computation time in five training sets for varying *n*, *p*, and *G.*

The MSE performance on the test sets for all methods is shown for informative groups (Figure 3) and weakly informative or no groups (Figure B2, supplementary materials). Our proposed method performs well. The method squeezy (single) (either with or without reCV) outperforms the other methods in the single group setting. The difference in performance between squeezy (single + reCV) and EN is likely due to the different methods for determining the penalty, marginal likelihood versus CV. More importantly, squeezy (multi) (either with or without reCV) outperforms the other methods including squeezy (single) in the setting with informative groups, illustrating the benefit of informative co-data. The performance of squeezy (multi) and squeezy (single) is more alike in the setting with weakly informative groups, and superior to the other methods. The method performs better when maximum marginal likelihood estimates for ridge penalties are transformed (squeezy) than when moment estimates are transformed (ecpcEN squeezy). Note that the performance of ipf might improve by using a larger grid, but only at a very substantial computational cost as shown in Figure 4. While the difference in performance between squeezy and the other methods is smaller for α=0.8 , squeezy (multi) still outperforms the other methods when the co-data is informative (Figure B3 in the supplementary materials).

#### 3.1.2 Group Parameter Estimates

For *α* = 1, we may compare the empirical Bayes group parameter estimates *λ_g_* and *τ_g_* obtained from ecpcEN squeezy (marginal moment estimator) and squeezy (multi) (marginal likelihood estimator) with the “true” sample estimates obtained from maximizing the likelihood and lasso prior given the sampled *X*, ***Y*** and β in each dataset:

$$\begin{array}{c} \sigma^{*}{}^{2}=\frac{1}{n-1}\sum_{i=1}^{n}{\left(Y_{i}-X_{i}\beta \right)}^{2},\\ \tau_{g}^{*}{}^{2}=\frac{1}{2(p/G)}\sum_{\left\{k\in \{1,..,p\}:g_{k}=g\right\}}|\beta_{k}|,\;\lambda_{g}^{*}=\frac{\sigma^{*}{}^{2}}{\tau_{g}^{*}{}^{2}}. \end{array}$$

Figure B4, supplementary materials shows that squeezy estimates the group parameters well, while ecpcEN squeezy underestimates $\tau_{g}^{2}$ and overestimates *λ_g_*. This difference may be a result from jointly optimizing $\sigma^{2}$ and $\lambda_{R}$ in squeezy, while $\lambda_{R}$ is estimated given a fixed estimate of $\sigma^{2}$ in ecpcEN squeezy.

#### 3.1.3 Computation Time Comparison

Next, we fit the methods on five training sets to compare computation time for varying *n*, *p* and *G*, for α=0.3 . Results are similar for other α . The group parameters are set according to the setting with informative groups. We set (n,p,G)=(150,1200,5) . Then, we vary one of *n*, *p*, *G* while keeping the others fixed. Figure 4 shows the average computation time for fitting the models. Unsurprisingly, the group-adaptive methods are slower than the other methods, but squeezy scales substantially better than ipf in the number of groups.

#### 3.1.4 Benefit of Co-data Learning for Variable Selection

We include two linear regression scenarios to show how variable selection may benefit from having differential group prior penalties. Suppose that *n* = 100 and *p* = 400, from which $p^{*}=24$ “true” regression coefficients are nonzero and the rest are zero. With the first scenario we aim to show that co-data can help to discriminate the contribution of a relevant variable from a strongly correlated nonrelevant one. With the second scenario we aim to show how co-data can improve variable selection particularly for variables with weaker signal. We consider four co-data settings: (A) strong; (B) moderately strong; (C) non-informative; (D) no groups. Co-data settings A, B, and C are fit with squeezy and are compared to D, fit with glmnet, all for *α* = 1 (lasso). Details for the simulation set up are given in Section B.1.2, supplementary materials.

**Fig. 5:**
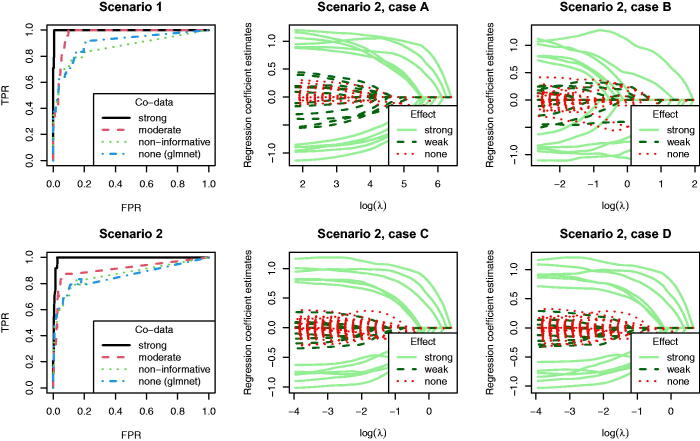
Model-based simulation study for variable selection, *α* = 1. Left column: ROC curves for variable selection for scenario 1 and 2, parameterized by the global L1-penalty. Middle and right column: traceplots for scenario 2 and cases A, B, C, and D. Continuous lines indicate p*/2 true variables with strong signal, dashed lines indicate p*/2 true variables with weak signal, dotted lines indicate the other variables with no signal.

Figure 5 shows the ROC curves for variable selection in both scenarios, parameterized by the global L1-penalty. Note that the models are not necessarily strictly nested, causing minor non-monotone effects. We observe clearly superior performance for squeezy in case of (moderately) strong co-data, and (near) equal performance to glmnet in case of non-informative co-data. For the first scenario, Figure B7, supplementary materials shows the trace plots for the four co-data settings. True variables come up earlier in both settings A and B, with strong and moderate co-data, respectively. Hence, variable selection performance is clearly better than for setting D, without co-data. In setting C, with nonrelevant co-data, variable selection performance is on par with that of setting D. The traceplots in Figure B8, supplementary materials highlight the 12 pairs of colinear variables for settings A and D. Overall, setting A better discriminates true from false variables. In particular, for variables 9 and 11 (first and third variable on bottom row) we observe that the no co-data setting tends to prefer the false variable, whereas squeezy prefers the true variable. For the second scenario, we observe that true variables come up earlier in both settings A and B, in particular for the weak, true variables (Figure 5). Hence, variable selection performance is clearly better than for setting D, without co-data. In setting C, with nonrelevant co-data, variable selection performance is on par with that of setting D.

So, variable selection improves markedly when accounting for strongly or moderately informative co-data. This improvement is particularly noticeable for true variables that are weaker either due to strong colinearity with a false variable (scenario 1), or due to a relatively small coefficient (scenario 2). It seems that the “weak” are helped by the “strong,” because they share co-data information.

### 3.2 Application to Predicting Therapy Response

We apply squeezy to predict therapy response (clinical benefit vs. progressed disease) using microRNA (miRNA) data from a study on colorectal cancer (Neerincx et al. 2018), consisting of *p* = 2114 miRNA expression levels for *n* = 88 independent individuals. As co-data, we use local false discovery rates (FDRs) for differential expression in the primary tumor versus adjacent colorectal tissue. The FDRs were obtained in a previous study (Neerincx et al. 2015) from a different set of nonoverlapping samples. These co-data were previously shown to be informative for the prediction, miRNAs that are tumor-specific tend to be more important for the response prediction (van Nee, Wessels, and van de Wiel 2021). Here, we discretise the continuous FDRs in *G* = 8 equally sized groups, such that we have around 10 samples per group parameter.

**Fig. 6:**
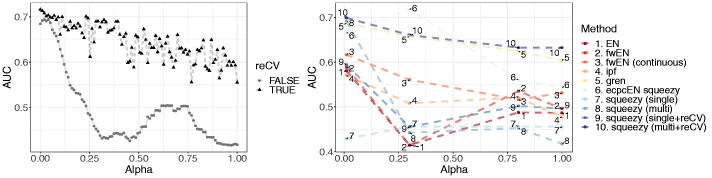
Results of 10-fold cross-validation in miRNA data example. Left: AUC performance for squeezy (multi) and squeezy (multi + reCV) for 100 evenly spaced values of α∈[0,1] . Right: AUC performance for several values of α for various methods.

We fit the methods listed above on 10-fold to compare cross-validated performance (Figure 6) and average computation time (Table 2). A visual check of the QQ-plot shows that the normality assumption of squeezy is reasonable (Figure B9, Supplementary materials). Figure 6 (left) clearly shows the benefit of recalibration by cross-validation (reCV) in this logistic regression setting. Our method squeezy (multi + reCV) performs as well as gren (Figure 6 (right), cf 10 vs. 5) but is around 25 times as fast. The method ecpcEN squeezy is twice as fast as squeezy (multi + reCV) and is competitive to squeezy (multi + reCV) and gren. These three methods all benefit from the informative co-data and outperform the other methods.

**Table 2:** Results of 10-fold CV in miRNA data example.

Method	Time (s)	Method	Time (sec)
1. EN	1.17 (1.06)	2. fwEN (groups)	12.20 (6.84)
7. squeezy (single)	5.12 (3.29)	10. squeezy (multi + reCV)	13.58 (13.59)
6. ecpcEN squeezy	5.48 (3.01)	3. fwEN (continuous)	14.11 (11.13)
9. squeezy (single + reCV)	7.45 (5.77)	5. gren	351.52 (96.74)
8. squeezy (multi)	10.47 (10.50)	4. ipf	422.39 (259.80)

NOTE: Mean time per fold and standard deviation over folds, sorted from shortest to longest time.

### 3.3 Application to Diagnostic Classification

We apply the method to methylation data with the goal of classifying samples as normal cervical tissue or precursor lesion, which has a high risk of progressing to cancer. The data consist of methylation levels for *p* = 40,000 probes in *n* = 37 samples and are extensively described in van de Wiel et al. (2016). The available co-data are biologically defined groups which are based on the distance to so-called CpG-islands. CpG islands are regions in the DNA that are often close to gene promotor regions and may therefore be more important for classification than other regions. The six groups ranging from close to far from an island are: CpG island, North Shore, South Shore, North Shelf, South Shelf, and Distant.

**Fig. 7:**
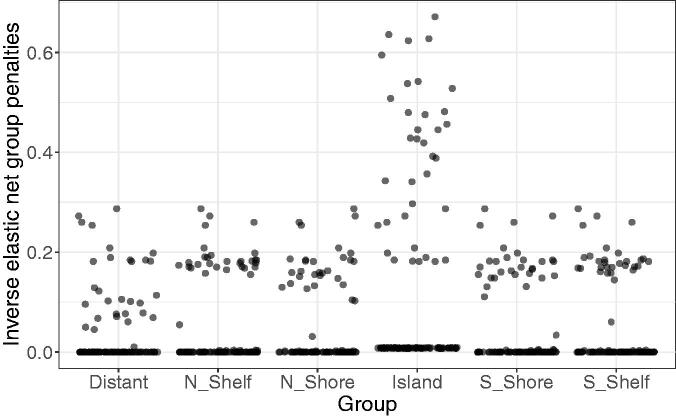
Methylation data example. Estimated inverse elastic net group penalties in all folds in ten 10-fold cross-validations of the data.

We fit an elastic net (α=0.3 ) logistic regression model with or without co-data and the methods described above in ten 10-fold cross-validations. A visual check of the QQ-plot shows that the normality assumption of squeezy is reasonable (Figure B10, supplementary materials). The inverse elastic net group penalties estimated with squeezy are relatively large for the CpG island group and relatively small for the Distant group (Figure 7), corroborating the biological expectations. Note that for some folds, the estimated inverse penalties are close to zero, corresponding to nearly full shrinkage of the regression coefficients. This may be due to the small sample size. Table 3 shows the results for the average computation time, AUC performance measure, number of selected variables and cross-validated log-likelihood (CVLL) for various methods. Recalibration by cross-validation (reCV) is essential for good performance of squeezy. When compared to the co-data agnostic elastic net model, the performance of our proposed method squeezy (multi + reCV) is similar in terms of AUC and slightly better in terms of the cross-validated log-likelihood. Our method squeezy (multi + reCV) is faster than the other group-adaptive methods and more memory-efficient than ipf2 and gren. The group-adaptive methods ipf2 and gren did not run on the full data due to memory issues and have therefore been fit on a smaller, random subsample of variables on which the methods were able to run; a subset of 5000 variables for ipf2 and one of 20,000 for gren.

**Table 3:** Results of ten 10-fold cross-validations in the methylation data example.

Method	Time (sec)	AUC	# **Variables**	CVLL
EN	2.88 (± 0.14)	0.938 (± 0.017)	182 (± 27)	−11.34 (± 1.50)
fwEN	74.66 (± 24.32)	0.935 (± 0.015)	198 (± 17)	−11.21 (± 1.09)
ipf	173.12 (± 18.50)	0.937 (± 0.015)	60 (± 29)	−16.38 (± 1.04)
ipf2${}^{*}$	473.65 (± 9.38)	0.902 (± 0.039)	59 (± 35)	−17.13 (± 1.80)
gren ${}^{*}$	32.88 (± 1.63)	0.933 (± 0.011)	167 (± 19)	−11.67 (± 0.86)
ecpcEN squeezy	80.77 (± 86.99)	0.839 (± 0.025)	129 (± 28)	−18.41 (± 1.72)
squeezy (single)	12.69 (± 9.48)	0.608 (± 0.039)	36 (± 55)	−22.90 (± 1.01)
squeezy (multi)	26.89 (± 21.35)	0.658 (± 0.059)	47 (± 63)	−21.62 (± 1.43)
squeezy (single + reCV)	16.87 (± 8.18)	0.938 (± 0.016)	203 (± 14)	−10.96 (± 1.03)
squeezy (multi + reCV)	29.30 (± 20.99)	0.939 (± 0.019)	178 (± 19)	−10.58 (± 1.09)

NOTE: The mean and standard deviation of the computation time, AUC, number of selected variables and cross-validated log-likelihood (CVLL) are shown for several methods. *The methods ipf2 and gren did not run due too memory issues. Results shown are based on a smaller, random subsample of variables for which the methods were able to run, 5000 for ipf2 and 20,000 for gren, whereas the other methods use all 40,000 variables.

## 4 Discussion

The proposed method, termed squeezy, computes fast marginal likelihood estimates for group-adaptive elastic net penalties. The method estimates those penalties by deducing them from group-adaptive ridge penalties, for which we derive a low-dimensional representation of the Taylor approximation of the marginal likelihood and its first derivative for generalized linear models, using results from Wood (2011). Squeezy implements this and uses BFGS optimization to find the penalties. An extension would be to also use the Hessian (Wood 2011), which may speed up the optimization.

An alternative implementation of our method uses the R-package mgcv (Wood 2011) to estimate the ridge penalties from the linear predictors, as detailed in (van de Wiel, van Nee, and Rauschenberger 2021). As mgcv does not allow for more variables than samples, this solution cannot include unpenalized variables. A fix to this is to include these as pre-estimated offsets. In the absence of unpenalized variables, we found that the two implementations provided similar results at comparable computing times. The alternative solution opens up for application of squeezy to a wider variety of models (provided that the elastic net counter part is also available) such as the penalized Cox model for survival data.

We showed that the marginal likelihood of ridge models also approximates the marginal likelihood of elastic net models, by using the asymptotic multivariate normality of the linear predictor. This result also holds for other priors with finite variance. We provided examples for a spike-and-slab prior and generalized normal prior. Another example is to use a hierarchical prior for shrinkage or selection on group level when the co-data includes many groups. The result does not hold for priors with infinite variance, such as the highly sparse horseshoe prior (Carvalho, Polson, and Scott 2009). Moreover, the practical use of our method for other relatively sparse priors should be tested.

The method transforms the ridge penalties to elastic net penalties by using the variance function of the elastic net prior. We showed in two data examples that it is beneficial to recalibrate the transformed elastic net penalties in logistic regression. Furthermore, the simulation study and data examples showed that our method is more scalable and faster than other group-adaptive methods, while it benefits from co-data and performs as well as or better than other (group-adaptive) methods. Aside from prediction, variable selection is an important aim of sparse methods. Therefore, we showed that in combination with informative co-data the use of squeezy benefits variable selection.

As we consider settings with high-dimensional data, the groups of variables are usually large. The approximation of the prior on the linear predictors benefits from this, as the multivariate normal result holds asymptotically in the number of variables. Should squeezy perform inferior to other methods, it may be useful to check whether this might be due to invalidity of the multivariate normal assumption for η=Xβ , for which we included a posterior check. This check may be used too for data settings in which *n* is of the same order of magnitude as *p*. Our approach may be used in these settings, but will have less benefit than in a high-dimensional setting, as the likelihood will be relatively more informative compared to the prior. For the low-dimensional setting in which *n* > *p*, our approach cannot be used as the assumption on the rank of *X* in Theorem 1 is then violated.

As our method is based on (marginal) likelihood, it in principle facilitates model selection in terms of the hyper-parameters (e.g., single group vs. multi-group) by the use of information criteria (Greven and Kneib 2010). To what extent which criterion is useful in our setting is a topic for further research.

The software is available in the R-package squeezy on CRAN. We provide scripts demonstrating the package and reproducing the results on https://github.com/Mirrelijn/squeezy.

## Supplementary Material

Supplemental Material

## Supplementary Materials

Appendix

The online Appendix contains Supplemental Section A with details of the method and Supplemental Section B with additional results and figures to the data examples (Appendix_squeezy.pdf, pdf file).

Code

This supplemental zip-folder contains the code for reproducing all results, explained in the corresponding README.txt files (Code_squeezy.zip, zip file).
