# Supplementary material for: Fast Marginal Likelihood Estimation of Penalties for Group-Adaptive Elastic Net
Source: J Comput Graph Stat. 2022 Nov 9;32(3):950–60. doi: 10.1080/10618600.2022.2128809 (PMC10511031; doi:10.1080/10618600.2022.2128809)
Supplement: Supplemental Material [file UCGS_A_2128809_SM1508.zip › Appendix_squeezy.pdf]

Appendix to “Fast marginal likelihood estimation of penalties for group-adaptive elastic net” published in the Journal of Computational and Graphical Statistics

Mirrelijn M. van Nee*, Tim van de Brug and Mark A. van de Wiel
Department of Epidemiology and Data Science, Amsterdam University Medical Centers, Amsterdam, The Netherlands

*The first author is supported by ZonMw TOP grant COMPUTE CANCER (40-00812-98-16012).

A Details of the method

We first provide details of the derivation of a low-dimensional representation of the Laplace approximation for the marginal likelihood for group-adaptive ridge generalized linear models and its first derivative as stated in Equations (6) and (8). Then we provide details for the linear and logistic regression. Lastly, we provide details on handling overlapping groups, the normality check and extensions to two other sparse priors.

A.1 Laplace approximate marginal likelihood for group-adaptive ridge models

When some variables are left unpenalized, we need to integrate over the penalized variables only. Wood (2011) forms an ortogonal basis U for the range space of ridge penalty matrix S. For group-adaptive ridge penalties, this orthogonal basis is simply given by the identity matrix, as S is diagonal with zeros for unpenalized variables. The proposed reparametrization of X and S, denoted by $\bar{X}$ and $\bar{S}$ in Wood (2011), boils down to simply taking the columns in X that correspond to the penalized variables and taking the columns and rows of the penalty matrix that correspond to the penalized variables. Hence, for our application we substitute $\bar{X}$ and $\bar{S}$ in the equations from Wood (2011) by X_{pen} and Λ_{pen} respectively.

The high-dimensional log-marginal likelihood approximation is given in Equation (5) in Wood (2011) and may be written as follows:

$$-\ell(\boldsymbol{\lambda}, \phi) \approx -\ell(\hat{\boldsymbol{\beta}}, \phi) + \frac{1}{2\phi}\hat{\boldsymbol{\beta}}^T \Lambda \hat{\boldsymbol{\beta}} + \frac{1}{2}\log(|X_{pen}^T W X_{pen} + \Lambda_{pen}|) - \frac{1}{2}\log(|\Lambda_{pen}|). \tag{A1}$$

When we use Newton iterations to obtain $\hat{\boldsymbol{\beta}}$ or $\hat{\boldsymbol{\eta}}$, upon convergence:

$$\hat{\boldsymbol{\beta}} = (X^T W X + \Lambda)^{-1} X^T (\boldsymbol{y} - \boldsymbol{\mu} + W X \hat{\boldsymbol{\beta}}),$$
$$\hat{\boldsymbol{\eta}} = X (X^T W X + \Lambda)^{-1} X^T (\boldsymbol{y} - \boldsymbol{\mu} + W \hat{\boldsymbol{\eta}}).$$

We substitute this in the second term of the log-marginal likelihood approximation:

$$\begin{aligned}\hat{\boldsymbol{\beta}}^T\Lambda\hat{\boldsymbol{\beta}} &= (\boldsymbol{y}-\boldsymbol{\mu}+WX\hat{\boldsymbol{\beta}})^TX(X^TWX+\Lambda)^{-1}\Lambda\hat{\boldsymbol{\beta}}\\ &= (\boldsymbol{y}-\boldsymbol{\mu}+WX\hat{\boldsymbol{\beta}})^TX(I_{p\times p}-(X^TWX+\Lambda)^{-1}X^TWX)\hat{\boldsymbol{\beta}}\\ &= (\boldsymbol{y}-\boldsymbol{\mu}+WX\hat{\boldsymbol{\beta}})^TX\hat{\boldsymbol{\beta}}-(\boldsymbol{y}-\boldsymbol{\mu}+WX\hat{\boldsymbol{\beta}})^TX(X^TWX+\Lambda)^{-1}X^TWX\hat{\boldsymbol{\beta}}\\ &= (\boldsymbol{y}-\boldsymbol{\mu}+W\hat{\boldsymbol{\eta}})^T\hat{\boldsymbol{\eta}}-\hat{\boldsymbol{\eta}}^TW\hat{\boldsymbol{\eta}}\\ &= (\boldsymbol{y}-\boldsymbol{\mu})^T\hat{\boldsymbol{\eta}}.\end{aligned}$$

The latter two terms may be rewritten in a lower-dimensional representation by:

$$\begin{aligned}&\frac{1}{2}\log(|X_{pen}^TWX_{pen}+\Lambda_{pen}|)-\frac{1}{2}\log(|\Lambda_{pen}|)=-\frac{1}{2}\log(|(X_{pen}^TWX_{pen}+\Lambda_{pen})^{-1}\Lambda_{pen}|)\\ &=-\frac{1}{2}\log(|I_{pen\times pen}-(X_{pen}^TWX_{pen}+\Lambda_{pen})^{-1}X_{pen}^TWX_{pen}|)\\ &=-\frac{1}{2}\log(|I_{n\times n}-WX_{pen}(X_{pen}^TWX_{pen}+\Lambda_{pen})^{-1}X_{pen}^T|)\\ &=-\frac{1}{2}\log(|I_{n\times n}-WH_{pen}|),\end{aligned}$$

where efficient representations for H_{pen} exist (van de Wiel et al., 2021).

So, the low-dimensional Laplace approximation for the minus log-marginal likelihood is:

$$-\ell(\boldsymbol{\lambda},\phi)\approx-\ell(\hat{\boldsymbol{\eta}},\phi)+\frac{1}{2\phi}(\boldsymbol{y}-\boldsymbol{\mu})^T\hat{\boldsymbol{\eta}}-\frac{1}{2}\log(|I_{n\times n}-WH_{pen}|).$$

A.2 First derivative of Laplace approximate marginal likelihood for group-adaptive ridge models

We derive low-dimensional representations of the derivatives to $\rho_g=\log(\lambda_g)$ for $g=1,..,G$, as derived in a high-dimensional representation by Wood (2011).

A.2.1 Derivative of $\frac{\partial\hat{\boldsymbol{\eta}}}{\partial\rho_g}$

Recall that $\tilde{I}_g\in\mathbb{R}^{p\times p}$ denotes the diagonal matrix with diagonal element k equal to 1 if k is in group g and 0 otherwise. The derivative for $\hat{\boldsymbol{\beta}}$ is then given by Wood (2011). After

substituting S by Λ and S_g by $\tilde{I}_g$ we obtain:

$$\begin{aligned}
\frac{\partial \hat{\boldsymbol{\beta}}}{\partial \rho_g} &= -\exp(\rho_g)(X^T W X + S)^{-1} S_g \hat{\boldsymbol{\beta}} \\
&= -\exp(\rho_g)(X^T W X + \Lambda)^{-1} \tilde{I}_g \hat{\boldsymbol{\beta}} \\
&= -\exp(\rho_g)(X^T W X + \Lambda)^{-1} \Lambda \frac{1}{\lambda_g} \tilde{I}_g \hat{\boldsymbol{\beta}} \\
&= -\exp(\rho_g) \left[I_{p\times p} - (X^T W X + \Lambda)^{-1} X^T W X \right] \frac{1}{\lambda_g} \tilde{I}_g \hat{\boldsymbol{\beta}} \\
&= -\exp(\rho_g) \left[I_{p\times p} - (X^T W X + \Lambda)^{-1} X^T W X \right] \frac{1}{\lambda_g} \tilde{I}_g (X^T W X + \Lambda)^{-1} X^T \left(\boldsymbol{y} - \boldsymbol{\mu} + W \hat{\boldsymbol{\eta}} \right).
\end{aligned}$$

We can write the derivative for $\hat{\boldsymbol{\eta}}$ as:

$$\begin{aligned}
\frac{\partial \hat{\boldsymbol{\eta}}}{\partial \rho_g} &= X \frac{\partial \hat{\boldsymbol{\beta}}}{\partial \rho_g} \\
&= -\exp(\rho_g) X \left[I_{p\times p} - (X^T W X + \Lambda)^{-1} X^T W X \right] \frac{1}{\lambda_g} \tilde{I}_g (X^T W X + \Lambda)^{-1} X^T \left(\boldsymbol{y} - \boldsymbol{\mu} + W \hat{\boldsymbol{\eta}} \right) \\
&= -\exp(\rho_g) \left[I_{n\times n} - X (X^T W X + \Lambda)^{-1} X^T W \right] X \frac{1}{\lambda_g} \tilde{I}_g (X^T W X + \Lambda)^{-1} X^T \left(\boldsymbol{y} - \boldsymbol{\mu} + W \hat{\boldsymbol{\eta}} \right) \\
&= -\exp(\rho_g) \left[I_{n\times n} - H W \right] \frac{1}{\lambda_g} H_g^T \left(\boldsymbol{y} - \boldsymbol{\mu} + W \hat{\boldsymbol{\eta}} \right),
\end{aligned}$$

with H_g, which can be seen as a contribution of the gth group to the hat matrix, defined as:

$$\begin{aligned}
H_g := X (X^T W X + \Lambda)^{-1} \tilde{I}_g X^T = W^{-\frac{1}{2}} P_1 W^{\frac{1}{2}} \Big(& I_{n\times n} - X_{pen} \Lambda_{pen}^{-1} X_{pen}^T W^{\frac{1}{2}} P_1 W^{-\frac{1}{2}} \\
& \cdot [W^{-1} + X_{pen} \Lambda^{-1} X_{pen}^T W^{\frac{1}{2}} P_1 W^{-\frac{1}{2}}]^{-1} \Big) \lambda_g^{-1} X_g X_g^T,
\end{aligned}$$

where we have used (van de Wiel et al., 2021):

$$\begin{aligned}
P_1 &= I_{n\times n} - W^{\frac{1}{2}} X_{unpen} (X_{unpen}^T W X_{unpen})^{-1} X_{unpen}^T W^{\frac{1}{2}}, \\
[X (X^T W X + \Lambda)^{-1}]_{pen} &= W^{-\frac{1}{2}} P_1 W^{\frac{1}{2}} \Big(X_{pen} \Lambda_{pen}^{-1} - X_{pen} \Lambda_{pen}^{-1} X_{pen}^T W^{\frac{1}{2}} P_1 W^{-\frac{1}{2}} \\
& \quad \cdot [W^{-1} + X_{pen} \Lambda^{-1} X_{pen}^T W^{\frac{1}{2}} P_1 W^{-\frac{1}{2}}]^{-1} X_{pen} \Lambda_{pen}^{-1} \Big).
\end{aligned}$$

A.2.2 Derivative of $\frac{\partial \ell(\hat{\boldsymbol{\eta}}, \phi)}{\partial \rho_g}$

Wood (2008) derives partials for the deviance of generalized linear models:

$$\frac{\partial \ell(\hat{\boldsymbol{\beta}}, \phi)}{\partial \hat{\boldsymbol{\beta}}} = \frac{1}{\phi} X^T \frac{\boldsymbol{y} - \boldsymbol{\mu}}{V(\boldsymbol{\mu}) \odot g'(\boldsymbol{\mu})},$$

where division by $V(\boldsymbol{\mu}) \odot g'(\boldsymbol{\mu})$ is element-wise and $\odot$ representing element-wise multiplication. The derivative of the log likelihood is then given by:

$$
\begin{aligned}
\frac{\partial \ell(\hat{\boldsymbol{\eta}}, \phi)}{\partial \rho_g} = \frac{\partial \ell(\hat{\boldsymbol{\beta}}, \phi)}{\partial \rho_g} &= \frac{\partial \ell(\hat{\boldsymbol{\beta}}, \phi)}{\partial \hat{\boldsymbol{\beta}}}^T \frac{\partial \hat{\boldsymbol{\beta}}}{\partial \rho_g} \\
&= -\exp(\rho_g) \frac{1}{\phi} \left(\frac{\boldsymbol{y} - \boldsymbol{\mu}}{V(\boldsymbol{\mu})} \right)^T G'^{-1} X (X^T W X + \Lambda)^{-1} \tilde{I}_g \hat{\boldsymbol{\beta}} \\
&= -\exp(\rho_g) \frac{1}{\phi} \left(\frac{\boldsymbol{y} - \boldsymbol{\mu}}{V(\boldsymbol{\mu})} \right)^T G'^{-1} \left[I_{n \times n} - HW \right] \frac{1}{\lambda_g} H_g^T \left(\boldsymbol{y} - \boldsymbol{\mu} + W \hat{\boldsymbol{\eta}} \right),
\end{aligned}
$$

with G' a diagonal matrix with diagonal elements $g'(\mu_i)$.

A.2.3 Derivative of $\frac{1}{2\phi}(\boldsymbol{y} - \boldsymbol{\mu})^T \hat{\boldsymbol{\eta}}$

We have for the low-dimensional penalty term, recall that $\boldsymbol{\mu} = g^{-1}(\boldsymbol{\eta})$:

$$
\begin{aligned}
\frac{\partial}{\partial \rho_g} \left(\frac{1}{2\phi} (\boldsymbol{y} - \boldsymbol{\mu})^T \hat{\boldsymbol{\eta}} \right) &= \frac{1}{2\phi} \left(\frac{\partial (\boldsymbol{y} - \boldsymbol{\mu})^T}{\partial \rho_g} \hat{\boldsymbol{\eta}} + (\boldsymbol{y} - \boldsymbol{\mu})^T \frac{\partial \hat{\boldsymbol{\eta}}}{\partial \rho_g} \right) \\
&= \frac{1}{2\phi} \left(\frac{\partial (-\boldsymbol{\mu}^T)}{\partial \rho_g} \hat{\boldsymbol{\eta}} + (\boldsymbol{y} - \boldsymbol{\mu})^T \frac{\partial \hat{\boldsymbol{\eta}}}{\partial \rho_g} \right) \\
&= \frac{1}{2\phi} \left(-\frac{\partial \hat{\boldsymbol{\eta}}^T}{\partial \rho_g} G'^{-1} \hat{\boldsymbol{\eta}} + (\boldsymbol{y} - \boldsymbol{\mu})^T \frac{\partial \hat{\boldsymbol{\eta}}}{\partial \rho_g} \right) \\
&= \frac{1}{2\phi} \left(-G'^{-1} \hat{\boldsymbol{\eta}} + \boldsymbol{y} - \boldsymbol{\mu} \right)^T \frac{\partial \hat{\boldsymbol{\eta}}}{\partial \rho_g} \\
&= -\exp(\rho_g) \frac{1}{2\phi} \left(-G'^{-1} \hat{\boldsymbol{\eta}} + \boldsymbol{y} - \boldsymbol{\mu} \right)^T \left[I_{n \times n} - HW \right] \frac{1}{\lambda_g} H_g^T \left(\boldsymbol{y} - \boldsymbol{\mu} + W \hat{\boldsymbol{\eta}} \right).
\end{aligned}
$$

A.2.4 Derivative of $-\frac{1}{2}\log(|\boldsymbol{I}_{n\times n} - \boldsymbol{W}\boldsymbol{H}_{pen}|)$

We have for the third term in Equation (A1):

$$\begin{aligned}
\frac{\partial}{\partial \rho_g}\left(\frac{1}{2}\log(|X_{pen}^T W X_{pen} + \Lambda_{pen}|\right) &= \frac{1}{2}tr\left((X_{pen}^T W X_{pen} + \Lambda_{pen})^{-1}\frac{\partial\left(X_{pen}^T W X_{pen} + \Lambda_{pen}\right)}{\partial \rho_g}\right) \\
&= \frac{1}{2}tr\left((X_{pen}^T W X_{pen} + \Lambda_{pen})^{-1} X_{pen}^T \frac{\partial W}{\partial \rho_g} X_{pen}\right) \\
&\quad + \frac{1}{2}tr\left((X_{pen}^T W X_{pen} + \Lambda_{pen})^{-1}\exp(\rho_g)\tilde{I}_g\right) \\
&= \frac{1}{2}tr\left(X_{pen}(X_{pen}^T W X_{pen} + \Lambda_{pen})^{-1} X_{pen}^T \frac{\partial W}{\partial \rho_g}\right) \\
&\quad + \frac{1}{2}tr\left((X_{pen}^T W X_{pen} + \Lambda_{pen})^{-1}\exp(\rho_g)\tilde{I}_g\right) \\
&= \frac{1}{2}tr\left(H_{pen}\frac{\partial W}{\partial \rho_g}\right) + \frac{1}{2}tr(\tilde{I}_g) \\
&\quad - \frac{1}{2}tr\left(\Lambda_{pen}^{-1}X_{pen}^T(W^{-1} + X_{pen}\Lambda_{pen}^{-1}X_{pen}^T)^{-1}X_{pen}\tilde{I}_g\right) \\
&= \frac{1}{2}tr\left(H_{pen}\frac{\partial W}{\partial \rho_g}\right) + \frac{1}{2}tr(\tilde{I}_g) \\
&\quad - \frac{1}{2}tr\left(\lambda_g^{-1}X_g X_g^T(W^{-1} + X_{pen}\Lambda_{pen}^{-1}X_{pen}^T)^{-1}\right),
\end{aligned}$$

where we have used the following equalities:

$$\begin{aligned}
(X_{pen}^T W X_{pen} + \Lambda_{pen})^{-1} &= \Lambda_{pen}^{-1} - \Lambda_{pen}^{-1}X_{pen}^T(W^{-1} + X_{pen}\Lambda_{pen}^{-1}X_{pen}^T)^{-1}X_{pen}\Lambda_{pen}^{-1} \\
H_{pen} &= X_{pen}(X_{pen}^T W X_{pen} + \Lambda_{pen})^{-1}X_{pen}^T,
\end{aligned}$$

with the partials of $W \in \mathbb{R}^{n\times n}$ readily obtained from Wood (2011).

For the latter term in Equation (A1):

$$\begin{aligned}
\frac{\partial}{\partial \rho_g}\left(-\frac{1}{2}\log(|\Lambda_{pen}|)\right) &= -\frac{1}{2}tr\left(\Lambda_{pen}^{-1}\frac{\partial \Lambda_{pen}}{\partial \rho_g}\right) \\
&= -\frac{1}{2}tr\left(\Lambda_{pen}^{-1}\tilde{I}_g\frac{\partial \lambda_g}{\partial \rho_g}\right) \\
&= -\frac{1}{2}tr(\tilde{I}_g)\frac{1}{\lambda_g}\lambda_g \\
&= -\frac{1}{2}tr(\tilde{I}_g).
\end{aligned}$$

So, the derivative of the Laplace approximate minus log marginal likelihood is given by:

$$\begin{aligned}\frac{\partial\left(-\ell(\boldsymbol{\rho},\phi)\right)}{\partial\rho_g} &= \frac{1}{\phi}\left(\frac{\boldsymbol{y}-\boldsymbol{\mu}}{V(\boldsymbol{\mu})}\right)^T G'^{-1}\left[I_{n\times n}-HW\right]H_g^T\left(\boldsymbol{y}-\boldsymbol{\mu}+W\hat{\boldsymbol{\eta}}\right)\\ &\quad-\frac{1}{2\phi}\left(-G'^{-1}\hat{\boldsymbol{\eta}}+\boldsymbol{y}-\boldsymbol{\mu}\right)^T\left[I_{n\times n}-HW\right]H_g^T\left(\boldsymbol{y}-\boldsymbol{\mu}+W\hat{\boldsymbol{\eta}}\right)\\ &\quad+\frac{1}{2}tr\left(H_{pen}\frac{\partial W}{\partial\rho_g}\right)-\frac{1}{2}tr\left(\lambda_g^{-1}X_gX_g^T(W^{-1}+X_{pen}\Lambda_{pen}^{-1}X_{pen}^T)^{-1}\right).\end{aligned}$$

A.3 Details for linear regression

Denote by $\varphi(\cdot;\mu,\sigma^2)$ the standard normal probability density function with mean μ and variance σ^2. The Laplace approximation is in fact exact for linear regression. We need the following parameters:

$$\phi=\sigma^2, V(\mu)=1, \ell(\hat{\boldsymbol{\eta}},\sigma^2)=\sum_{i=1}^{n}\log(\varphi(y_i;\hat{\boldsymbol{\eta}}_i,\sigma^2)),$$

$$\boldsymbol{\mu}=\hat{\boldsymbol{\eta}},\ W=I_{n\times n},\ g(\mu)=\mu,\ g'(\mu)=1,\ G'^{-1}=I_{n\times n}.$$

The partial derivative of the minus log likelihood to the scale parameter $\phi=\sigma^2$ from Equation (9) is given by:

$$\frac{\partial\left(-\ell(\hat{\boldsymbol{\eta}},\sigma^2)\right)}{\partial\sigma^2}=\frac{n}{2\sigma^2}-\frac{1}{2\sigma^4}(\boldsymbol{y}-\hat{\boldsymbol{\eta}})^T(\boldsymbol{y}-\hat{\boldsymbol{\eta}}).$$

A.4 Details for logistic regression

We need the following parameters:

$$\phi=1, V(\mu_i)=\mu_i(1-\mu_i), \ell(\hat{\boldsymbol{\eta}})=\sum_{i=1}^{n}y_i\log(expit(\hat{\eta}_i))+(1-y_i)\log(1-expit(\hat{\eta}_i)),$$

$$\boldsymbol{\mu}=expit(\hat{\boldsymbol{\eta}})=(1+\exp(-\hat{\boldsymbol{\eta}}))^{-1},$$

$$W=diag(\boldsymbol{\mu}\odot(1-\boldsymbol{\mu})),\ \alpha_i=1,\ \frac{\partial w_i}{\partial\eta_i}=\mu_i(1-\mu_i)(2\mu_i-1),$$

$$g(\mu_i)=logit(\mu_i),\ g'(\mu_i)=(\mu_i(1-\mu_i))^{-1},\ G'^{-1}=W,\ g''(\mu_i)=\frac{2\mu_i-1}{\mu_i^2(1-\mu_i)^2}.$$

A.5 Overlapping groups

Our method allows for partly overlapping groups by making artifical, non-overlapping groups. First consider group-regularized ridge models with group prior variances $\boldsymbol{\tau}_R^2 = \phi\boldsymbol{\lambda}_R^{-1}$. We model the prior variance by the average over multiple groups, as used in van Nee et al. (2021). Let the co-data matrix $Z \in \mathbb{R}^{p\times G}$ contain group membership information: for non-overlapping groups, Z_{kg} is 1 if variable k belongs to group g and 0 otherwise. For overlapping groups, we account for multiplicity by dividing each row k by the number of groups variable k belongs to, i.e. for variable k that belongs to multiple groups given in $\mathcal{I}_k \subseteq \{1, .., G\}$, we set $Z_{kg} = 1/|\mathcal{I}_k|$ for $g \in \mathcal{I}_k$ and 0 otherwise (van Nee et al., 2021). The vector with averaged prior variances for each variable is then given by $Z\boldsymbol{\tau}_R^2$.

Let $\tilde{X}$ denote the observed data matrix where each column k of the original matrix X is duplicated $|\mathcal{I}_k|$ times for the number of groups variable k belongs to, with group indices given in $\mathcal{I}_k$. Let $\tilde{X}'$ denote the matrix where the columns are additionally scaled by $\frac{1}{\sqrt{|\mathcal{I}_k|}}$. Define the extended vector of artificial regression coefficients by $\tilde{\boldsymbol{\beta}}$ and the extended vector with scaled artificial regression coefficients by $\tilde{\boldsymbol{\beta}}'$. Each column duplicated from the original column k now corresponds to an artificial, independent effect $\tilde{\beta}_{k_g} = \frac{1}{\sqrt{|\mathcal{I}_k|}}\tilde{\beta}'_{k_g} \overset{ind.}{\sim} N(0, \tau_{R,g}^2/|\mathcal{I}_k|)$ for $k = 1, .., p$, and $g \in \mathcal{I}_k$. The effect of a variable k is equal to the sum of the contributions of the groups, $\beta_k = \sum_{g\in\mathcal{I}_k} \tilde{\beta}_{k_g} = \frac{1}{\sqrt{|\mathcal{I}_k|}} \sum_{g\in\mathcal{I}_k} \tilde{\beta}'_{k_g}$, such that $X\boldsymbol{\beta} = \tilde{X}\tilde{\boldsymbol{\beta}} = \tilde{X}'\tilde{\boldsymbol{\beta}}'$. The prior distribution of $\tilde{\beta}'_{k_g}$ is given by $N(0, \tau_{R,g}^2) = N(0, \phi\lambda_{R,g})$. Hence, we can use our proposed method on the scaled, duplicated $\tilde{X}'$ to obtain the estimates for the prior parameters $\phi, \boldsymbol{\lambda}_R$. Finally, the variance estimates are pooled by the co-data matrix Z to compute $\boldsymbol{\beta}$ with ridge prior variances $Z\boldsymbol{\tau}_R^2$.

For elastic net models, we first transform the ridge prior variances given in $Z\boldsymbol{\tau}_R^2$ to elastic net penalties as described in the main document.

A.6 Normality check

Figure A1 shows some example QQ-plots resulting from the normality check described in Section 2.6 for the ridge, lasso and horseshoe prior (Carvalho et al., 2009). The ridge and lasso prior have finite variance, while the horseshoe prior does not, thereby violating the

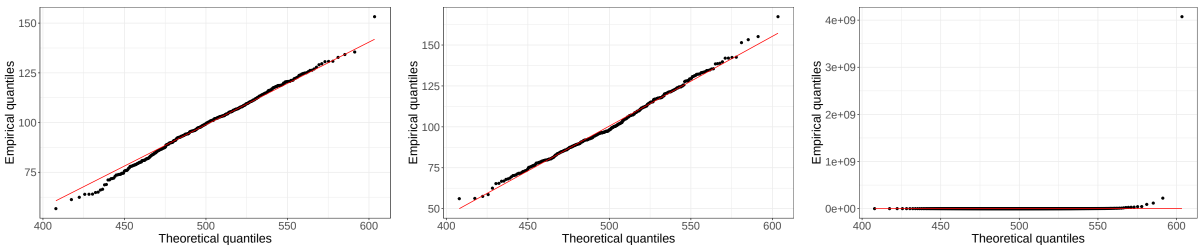

(a) Ridge (b) Lasso (c) Horseshoe

Figure A1: Example of QQ-plots obtained with the normality check described in Section 2.6. Elements of the data matrix $X \in \mathbb{R}^{100\times 300}$ are generated i.i.d. from a uniform distribution on $[0, 1]$. Regression coefficients are generated from a ridge prior (left), lasso prior (middle) or horseshoe prior (right). The theoretical and empirical quantiles align well for the ridge and lasso prior. Contrarily, they do not align well for the horseshoe prior, for which the finite prior variance assumption from Theorem 1 is violated.

assumptions from Theorem 1. Accordingly, the empirical and theoretical quantiles align well for the ridge and lasso prior, but do not align for the horseshoe prior.

A.7 Extension to other sparse priors

The method may be used to obtain group prior estimates for priors other than the group-adaptive elastic net prior. Below we give examples for the spike-and-slab prior and the generalized normal prior.

A.7.1 Spike-and-slab prior

Spike-and-slab priors differentiate between zero and non-zero effects by combining a spike with probability mass concentrated around 0 with a slab which is more diffused. Different types of models may be used for the spike and the slab part (Ishwaran et al., 2005; Ročková et al., 2018). Here, we consider the spike-and-slab prior for variable k in group g with a dirac spike and normal slab with group-specific inclusion probability $\theta_g \in [0, 1]$ and global slab prior variance $\tau^2_{slab} = \phi\lambda^{-1}_{slab}$:

$$\pi(\beta_k|\theta_g, \tau^2_{slab}) = (1 - \theta_g)\delta(\beta_k = 0) + \theta_g\varphi(\beta_k; 0, \tau^2_{slab}), \tag{A2}$$

with $\varphi(x; \mu, \sigma^2)$ denoting the probability density function of a normal distribution with mean μ and standard deviation σ. Note that the method does not allow for both a group-specific inclusion probability and a group-specific slab prior variance, used in Velten and Huber (2019), as the solution to Equation (A4) is then unidentifiable. Our choice here of having group-specific spikes and one global slab flexibly models the fraction of non-zero effects in groups and suits the purpose of variable selection. Alternatively, having group-specific slabs and one global spike may accommodate estimation of the non-zero effects well.

Usually, the global slab prior variance is not known. We propose to express the slab prior variance in terms of the global proportion of non-zero effects, θ_{global}. This parameter is then treated as tuning parameter similar to α in elastic net, and may be fixed at a prior guess or cross-validated. First, suppose that $\hat{\tau}^2_{R,global}$ is the global ridge prior variance corresponding to the situation in which all variables are grouped in one group. The variance function of the spike-and-slab prior and corresponding maximum marginal likelihood estimate for the slab prior variance are then given by:

$$h(\tau^2_{slab}) = \theta_{global}\tau^2_{slab},\ \hat{\tau}^2_{slab} = \hat{\tau}^2_{R,global}\theta^{-1}_{global}. \tag{A3}$$

Next, given the estimate for $\hat{\tau}^2_{slab}$, the variance function of the group-adaptive spike-and-slab and the corresponding marginal likelihood estimates for the inclusion probabilities are given by:

$$h(\theta_g) = \theta_g\tau^2_{slab},\ \hat{\boldsymbol{\theta}} = \hat{\boldsymbol{\tau}}^2_R\hat{\tau}^{-2}_{slab} = \theta_{global}\hat{\boldsymbol{\tau}}^2_R\hat{\tau}^{-2}_{R,global}, \tag{A4}$$

so the marginal likelihood estimates are simply the ratios between the ridge group prior variances and the global ridge prior variance, shrunk to 0 by the factor θ_{global}. Note that the ratios may be larger than 1 for some groups, for which the estimates may be truncated.

A.7.2 Generalized normal prior

The generalized normal prior corresponds to the bridge penalty (Frank and Friedman, 1993), which is an L_q penalty with $q > 0$ and includes the lasso penalty ($q = 1$) and ridge

penalty ($q = 2$). We consider the generalized normal prior for variable k in group g with group-specific penalty $\lambda_{b,g} > 0$ and scaled inverse penalties $\tau^2_{b,g} = \phi\lambda^{-1}_{b,g}$:

$$\pi(\beta_k|q, \lambda_{b,g}, \phi) = \frac{q\lambda_{b,g}^{1/q}}{2(2\phi)^{1/q}\Gamma(\frac{1}{q})} \exp\left(-\frac{\lambda_{b,g}}{2\phi}|\beta_k|^q\right), \tag{A5}$$

with $\Gamma(\cdot)$ the gamma function. Note that the group-specific penalties are scaled by 2ϕ such that the prior is consistent with the elastic net prior for $q = 2$ and $\alpha = 0$, and for $q = 1$ and $\alpha = 1$. The bridge penalty selects parameters for $q \in (0, 1]$ and shrinks parameters for $q > 1$. For a fixed q, the prior variance function and corresponding marginal likelihood estimates are given by:

$$h(\tau^2_{b,g}) = \frac{4^{1/q}\Gamma(\frac{3}{q})}{\Gamma(\frac{1}{q})}\tau_{b,g}^{4/q},\ \hat{\boldsymbol{\tau}}^2_b = \frac{1}{2}\left(\frac{\Gamma(\frac{1}{q})}{\Gamma(\frac{3}{q})}\right)^{\frac{q}{2}} \hat{\boldsymbol{\tau}}^q_R. \tag{A6}$$

The generalized normal prior has finite variance for $q > 0$. In practice, however, the prior variance may become large for q close to 0. One should take care whether the multivariate normal approximation is then sufficiently accurate for small $q > 0$. This may be checked a posteriori as described in Section 2.6.

B Additional results and figures to the data examples

B.1 Model-based simulation study

Figure B2 and B3 show the MSE performance in the model-based simulation study for $\alpha = 0.3$ and $\alpha = 0.8$ respectively. Figure B4 shows the group parameter estimates for linear regression.

B.1.1 Recalibration in logistic regression

We perform the same simulation study set up as in Section 3.1, but now consider binary response with logistic link function $Y \overset{ind.}{\sim} \text{Ber}(\text{expit}(X\boldsymbol{\beta}))$. Both the transformations of the ridge marginal moment estimates (`ecpcEN squeezy`) and Laplace approximation marginal likelihood estimates (`squeezy`) overestimate (underestimate) the penalty (prior variance)

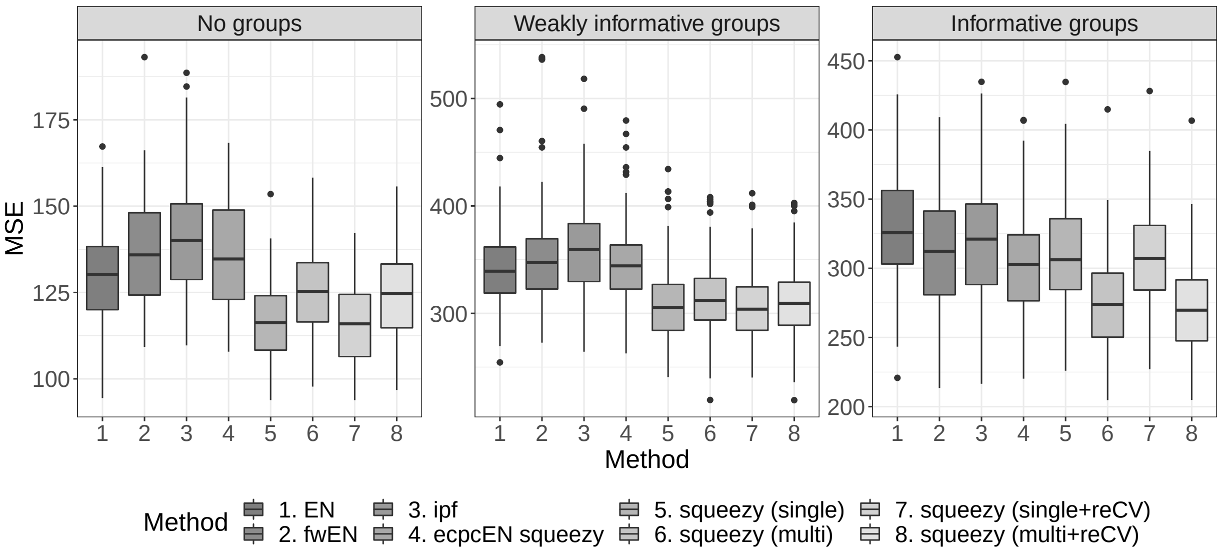

Figure B2: Model-based simulation study. Box plots of the MSE performance on the test sets of 100 pairs of training and test sets, $\alpha = 0.3$.

sample estimates (i.e. the maximum prior estimates given the known $\boldsymbol{\beta}$) (Figure B5). The group estimates are, however, estimated well in a relative sense: more important groups are penalized less, with larger differences between groups reflected in the group estimates (cf. setting `weakly informative groups` and `informative groups`, for example). Overpenalization of the logistic regression coefficients sometimes leads to intercept-only models, resulting in worse-than-random prediction performance in terms of AUC (AUC<0.5, Figure B6). Recalibrating the group penalties by cross-validation of a global penalty parameter counters the overpenalization and results in better prediction performance in terms of mean log likelihood on independent test sets (Figure B6). Note that while the sample group estimates best fit the prior given $\boldsymbol{\beta}$, these estimates are not necessarily the best for prediction purposes: directly using the sample estimates results in the best rank-based criterion AUC, but worst likelihood on the test samples. Hence, in contrast to the linear regression setting, in which the sample estimates could be viewed as "true" maximizers of the marginal likelihood, this is generally not the case for non-linear regressions which need to optimally trade off bias and variance for prediction purposes.

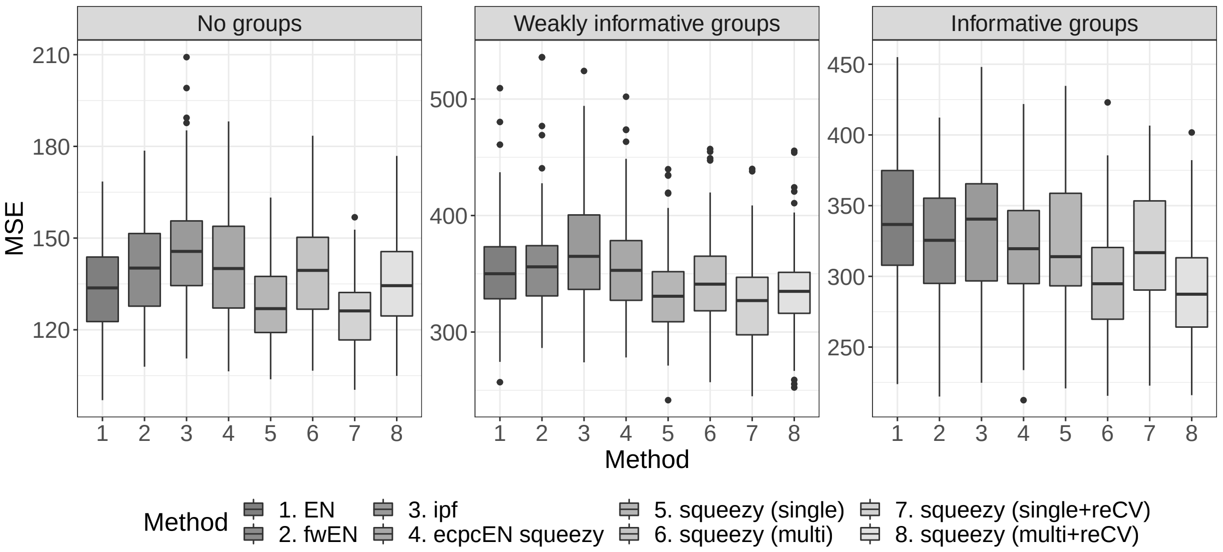

Figure B3: Model-based simulation study. Box plots of the MSE performance on the test sets of 100 pairs of training and test sets, $\alpha = 0.8$.

B.1.2 Details for simulation on variable selection

We have used the following simulation set up in the two scenarios used in Section 3.1.4:

1. Correlated variables: suppose that the first half of the true variables are strongly correlated with $p^*/2$ false variables with correlation coefficient $\rho = 0.8$, and the rest are standard normal:

$$\beta_j = (-1)^j,\ j = 1, .., p^*,\ \beta_j = 0,\ j = p^* + 1, .., p,$$
$$(X_{ij}, X_{i,p*+j}) \overset{ind.}{\sim} Biv - N(0, 1, \rho),\ i = 1, .., n,\ j = 1, .., p^*/2,$$
$$X_{ij} \overset{ind.}{\sim} N(0, 1),\ i = 1, .., n,\ j \in \{1, .., p\} \backslash \{1, .., p^*/2, p^* + 1, .., p^* + p^*/2\},$$
$$\boldsymbol{Y} \sim N(X\boldsymbol{\beta}, I_{n\times n}).$$

We consider four co-data settings: A) Strong co-data: group 1 contains all true variables and is complemented with p^* false variables, not including the ones colinear with true variables. Group 2 contains all other $p - 2p^*$ variables. So, these are strong co-data, as all true variables are in one group, but not perfect as group 1 is diluted by false variables; B) Moderate co-data: as A), but half of the true variables is moved to group 2. Note that (the smaller) group 1 is still over-represented with true variables;

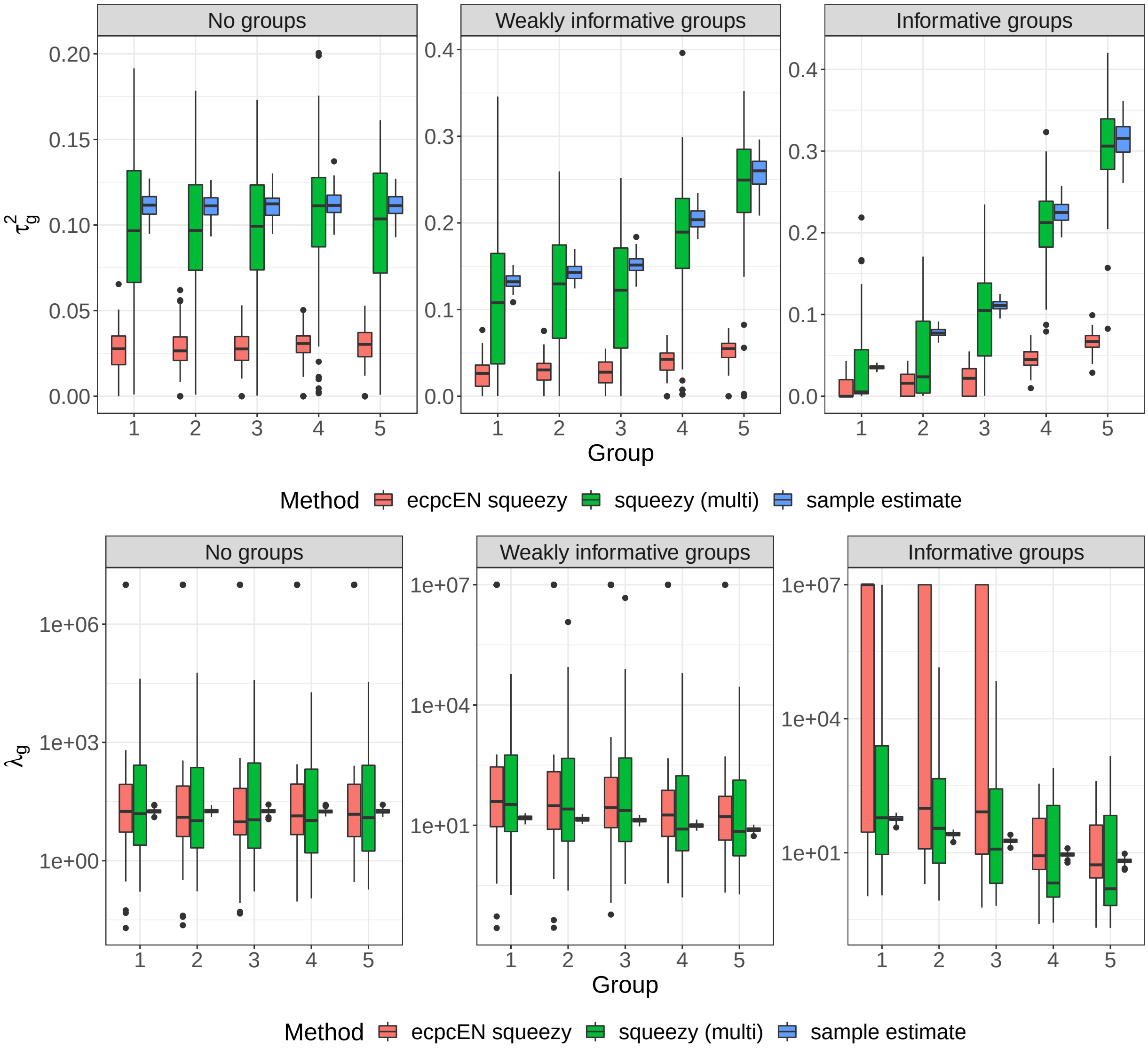

Figure B4: Model-based simulation study for linear regression. Group parameter estimates in 100 data sets for the lasso setting ($\alpha = 1$) compared to the sample estimates.

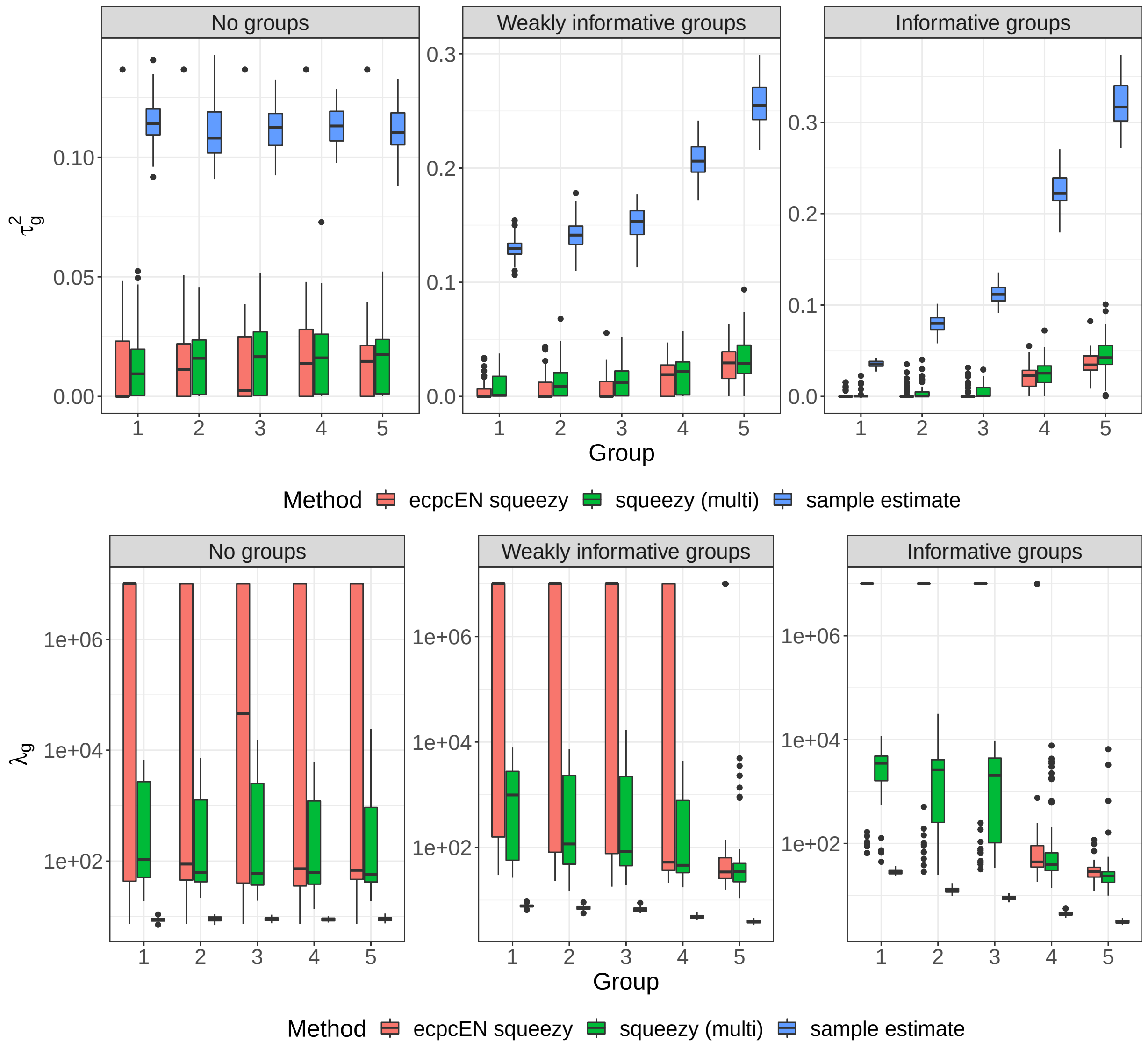

Figure B5: Model-based simulation study for logistic regression. Group parameter estimates without recalibration in 100 data sets for the lasso setting ($\alpha = 1$) compared to the sample estimates.

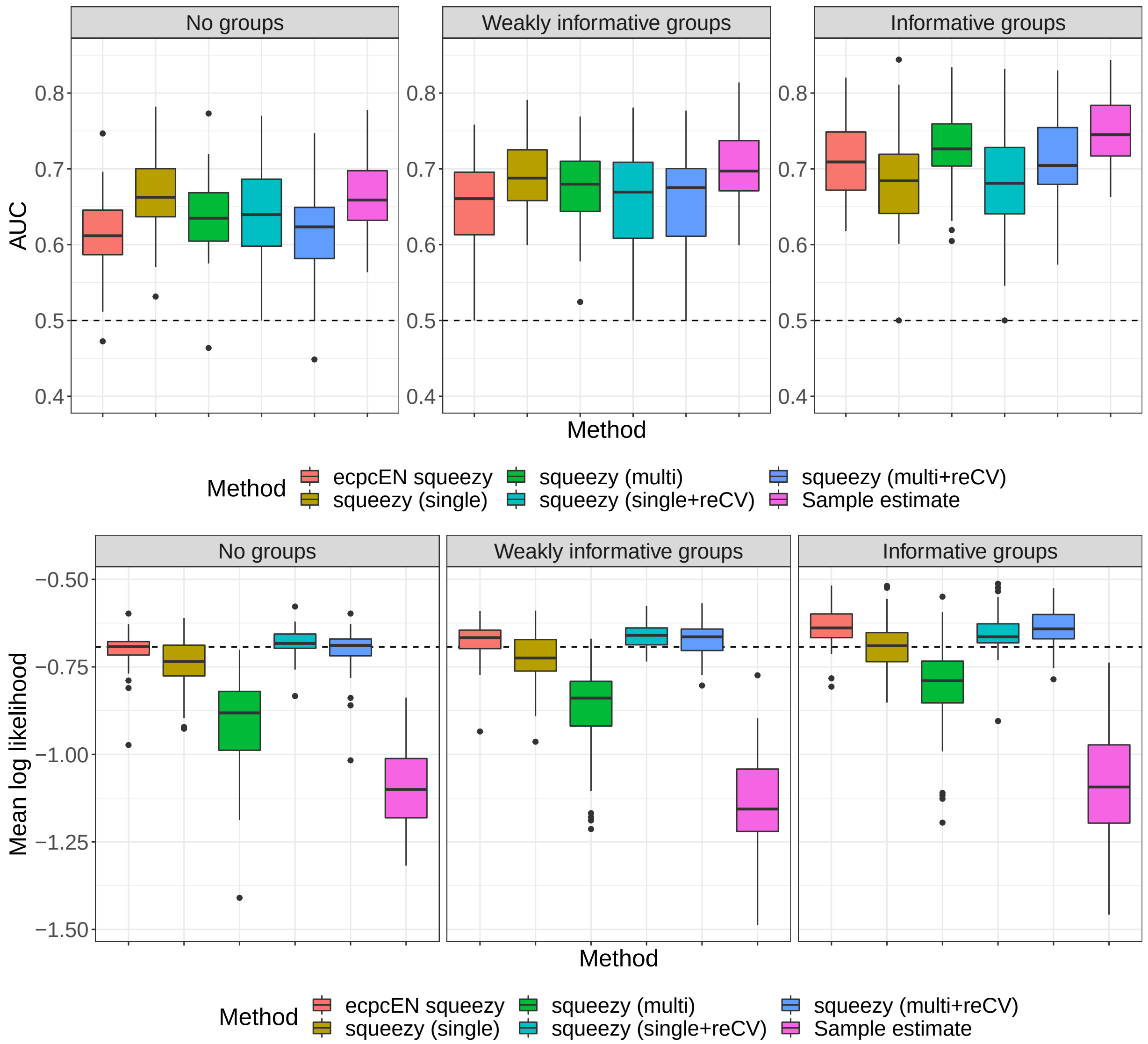

Figure B6: Model-based simulation study for logistic regression. Prediction performance measured on independent test data sets in terms of AUC and mean log likelihood when group parameters are estimated for the lasso setting ($\alpha = 1$) or when the sample estimates are used. Horizontal dashed line corresponds to the random guessing model.

C) Non-relevant co-data: more relevant variables are moved from group 1 to group 2, such that both contain the same proportions of true and false variables; D) No co-data.

2. Strong and weak signals: suppose that the first half of the true variables have a stronger signal than the second half of the true variables:

$$\beta_j = (-1)^j,\ j = 1, .., p^*/2,\ \beta_j = \frac{1}{3}(-1)^j,\ j = p^*/2, .., p^*,\ \beta_j = 0,\ j = p^* + 1, .., p,$$

$$X_{ij} \overset{i.i.d.}{\sim} N(0, 1),\ i = 1, .., n,\ j = 1, .., p,\ \boldsymbol{Y} \sim N(X\boldsymbol{\beta}, I_{n\times n}).$$

We consider the same four co-data settings as in the first scenario, where half of the true variables is randomly moved to group 2 in B).

Figure B7 shows the traceplots for the simulation study on variable selection in Scenario 1. Figure B8 highlights the traceplots of the 12 pairs of colinear variables.

B.2 Application to predicting therapy response

Figure B9 shows the QQ-plot for the normality check in the miRNA data.

B.3 Application to diagnostic classification

Figure B10 shows the QQ-plot for the normality check in the methylation data.

References

Carvalho, C. M., Polson, N. G., and Scott, J. G. (2009), "Handling sparsity via the horseshoe," in *Artificial Intelligence and Statistics*, PMLR.

Frank, I. E. and Friedman, J. H. (1993), "A statistical view of some chemometrics regression tools," *Technometrics*, 35, 109–135.

Ishwaran, H., Rao, J. S., et al. (2005), "Spike and slab variable selection: frequentist and Bayesian strategies," *Ann. Stat.*, 33, 730–773.

Ročková, V. et al. (2018), "Bayesian estimation of sparse signals with a continuous spike-and-slab prior," *Ann. Stat.*, 46, 401–437.

van de Wiel, M. A., van Nee, M. M., and Rauschenberger, A. (2021), "Fast cross-validation for multi-penalty high-dimensional ridge regression," *J. Comput. Graph. Stat.*, 1–13.

van Nee, M. M., Wessels, L. F., and van de Wiel, M. A. (2021), "Flexible co-data learning for high-dimensional prediction," *Stat. Med.*, 40, 5910–5925.

Velten, B. and Huber, W. (2019), "Adaptive penalization in high-dimensional regression and classification with external covariates using variational Bayes," *Biostatistics*. Kxz034.

Wood, S. N. (2008), "Fast stable direct fitting and smoothness selection for generalized additive models," *J. R. Stat. Soc. Ser. B Stat. Methodol.*, 70, 495–518.

— (2011), "Fast stable restricted maximum likelihood and marginal likelihood estimation of semiparametric generalized linear models," *J. R. Stat. Soc. Ser. B Stat. Methodol.*, 73, 3–36.

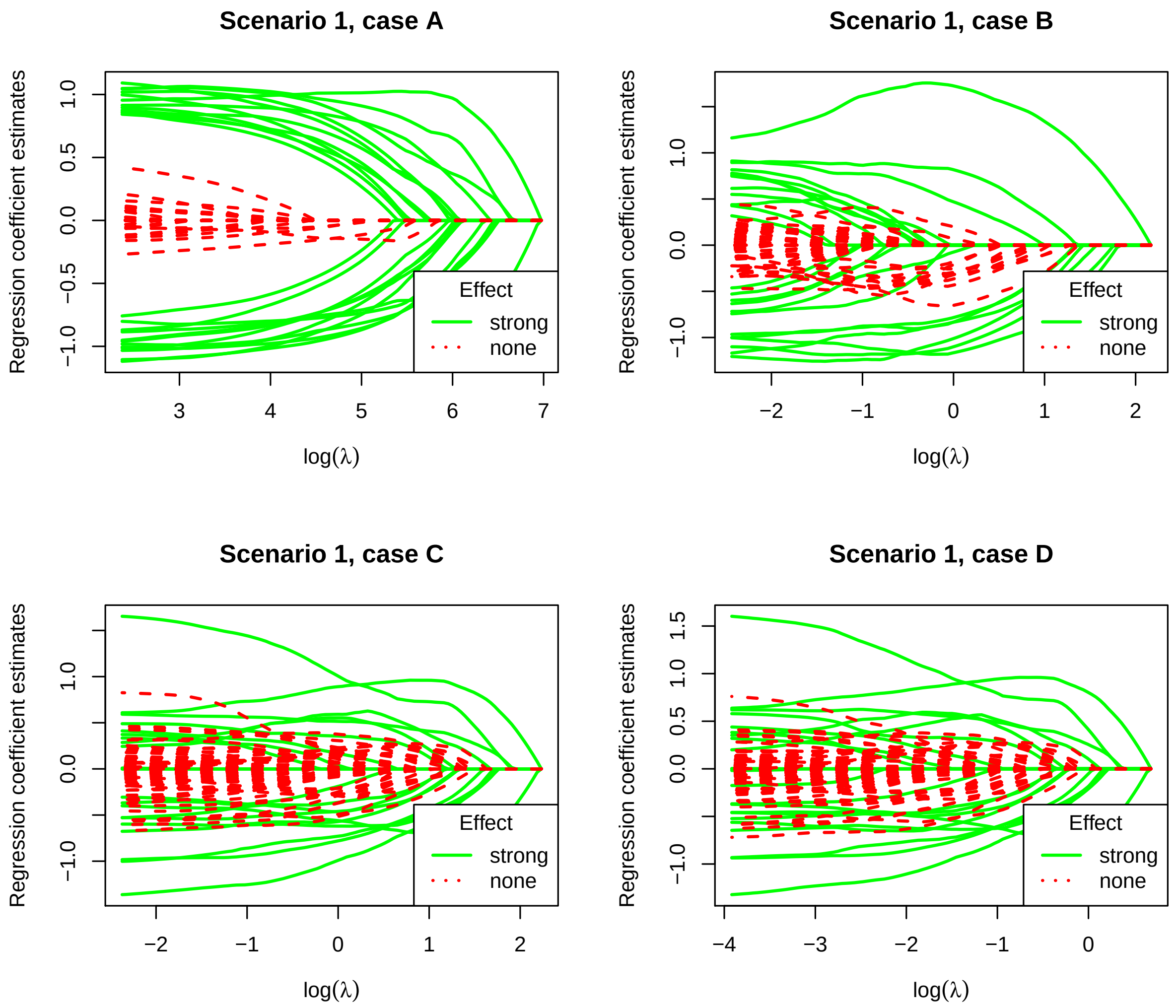

Figure B7: Traceplots for simulation study scenario 1 and cases A, B, C, and D. Lines indicate the p^* true variables, the dotted lines indicate the other variables.

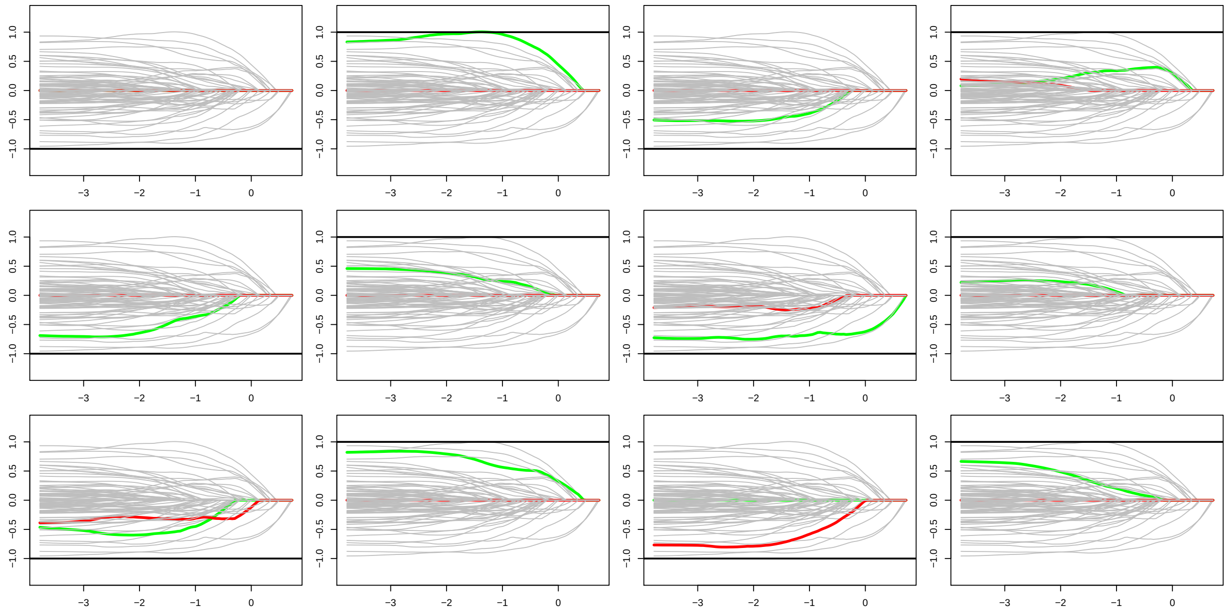

(a) `glmnet`

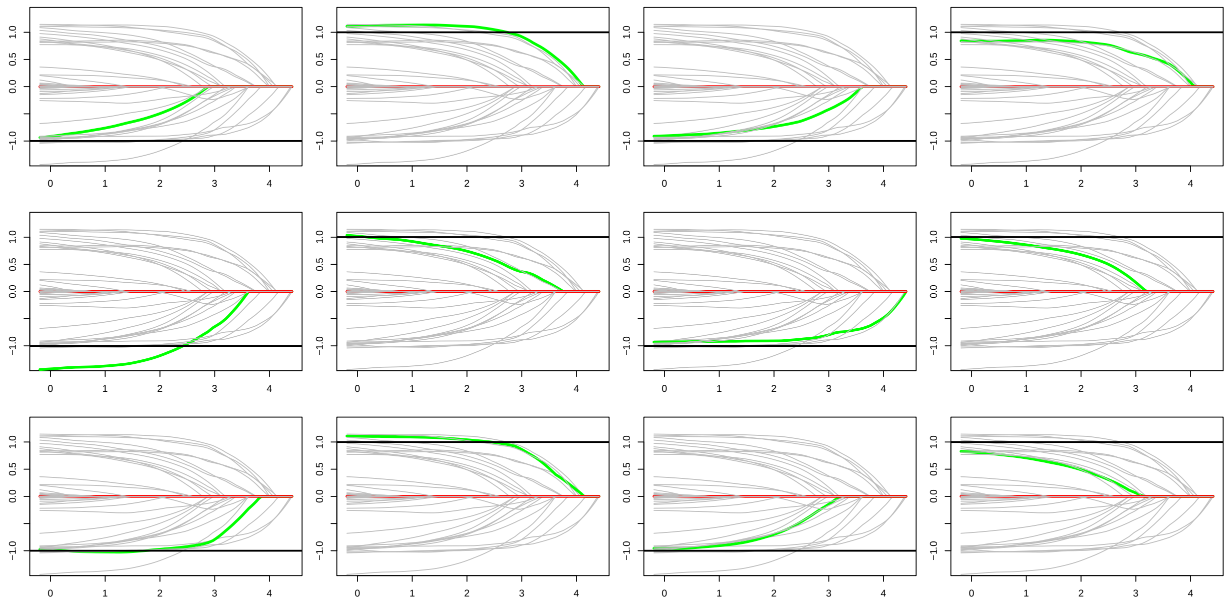

(b) `squeezy`

Figure B8: Results for simulation study scenario 1 with `glmnet` or `squeezy`. X-axis: log-lambda, y-axis: estimated coefficient. The traceplots highlight the 12 colinear variables for cases D and A. Green, bold lines: true variable; red, bold: colinear false variable. Black, horizontal lines: value of the true regression coefficient.

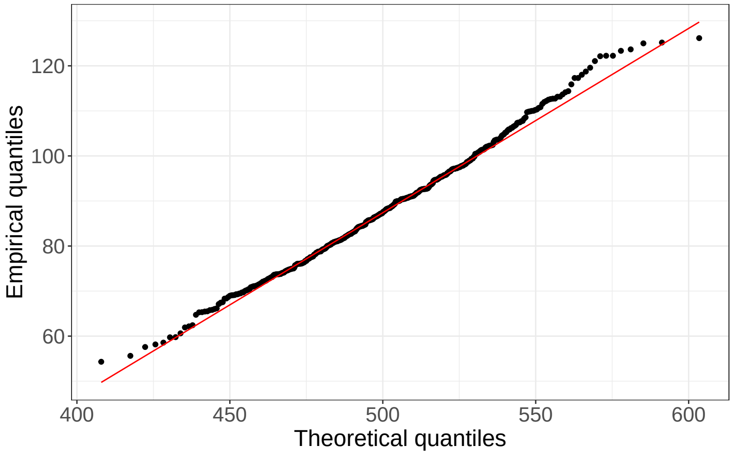

Figure B9: miRNA data example. QQ-plot for the normality check of the linear predictors.

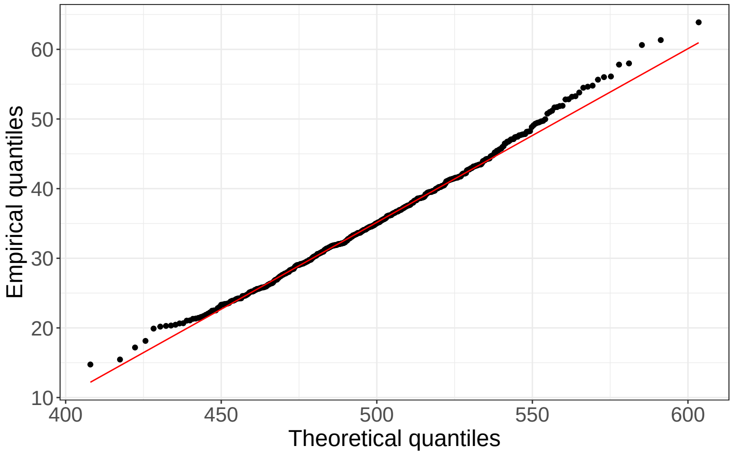

Figure B10: Methylation data example. QQ-plot for the normality check of the linear predictors.
